# Conditional Genetic Elimination of Hepatocyte Growth Factor in Mice Compromises Liver Regeneration after Partial Hepatectomy

**DOI:** 10.1371/journal.pone.0059836

**Published:** 2013-03-20

**Authors:** Kari Nejak-Bowen, Anne Orr, William C. Bowen, George K. Michalopoulos

**Affiliations:** Department of Pathology, University of Pittsburgh, Pittsburgh, Pennsylvania, United States of America; University of North Carolina School of Medicine, United States of America

## Abstract

Hepatocyte growth factor (HGF) has been shown to be indispensable for liver regeneration because it serves as a main mitogenic stimulus driving hepatocytes toward proliferation. We hypothesized that ablating HGF in adult mice would have a negative effect on the ability of hepatocytes to regenerate. Deletion of the HGF gene was achieved by inducing systemic recombination in mice lacking exon 5 of HGF and carrying the Mx1-cre or Cre-ER^T^ transgene. Analysis of liver genomic DNA from animals 10 days after treatment showed that a majority (70–80%) of alleles underwent cre-induced genetic recombination. Intriguingly, however, analysis by RT-PCR showed the continued presence of both unrecombined and recombined forms of HGF mRNA after treatment. Separation of liver cell populations into hepatocytes and non-parenchymal cells showed equal recombination of genomic HGF in both cell types. The presence of the unrecombined form of HGF mRNA persisted in the liver in significant amounts even after partial hepatectomy (PH), which correlated with insignificant changes in HGF protein and hepatocyte proliferation. The amount of HGF produced by stellate cells in culture was indirectly proportional to the concentration of HGF, suggesting that a decrease in HGF may induce de novo synthesis of HGF from cells with residual unrecombined alleles. Carbon tetrachloride (CCl4)-induced regeneration resulted in a substantial decrease in preexisting HGF mRNA and protein, and subsequent PH led to a delayed regenerative response. Thus, HGF mRNA persists in the liver even after genetic recombination affecting most cells; however, PH subsequent to CCl4 treatment is associated with a decrease in both HGF mRNA and protein and results in compromised liver regeneration, validating an important role of this mitogen in hepatic growth.

## Introduction

The partial hepatectomy (PH) model, in which two-thirds of the rat or mouse liver is removed and the remaining lobes enlarge to restore the original liver mass, is an ideal environment to study organ regeneration and controlled growth after injury [1], [2]. The hallmark of liver regeneration is proliferation of adult hepatic cell types, including hepatocytes, biliary epithelial cells, endothelial cells, and hepatic stellate cells (HSCs) [1]. The first peak of DNA synthesis occurs in hepatocytes around 24 hours in the rat and approximately 36 hours in the mouse [2]. Cell proliferation and regeneration is tightly regulated by a series of cell signaling pathways and cascades that are activated immediately after resection. One of these is the hepatocyte growth factor (HGF)/Met pathway.

HGF is a pleiotropic growth factor that has been shown to be essential for liver regeneration because it serves as the main mitogenic stimulus driving hepatocytes toward proliferation [1], [2]. Within minutes after PH, HGF is converted by urokinase plasminogen activator (uPA) to its active form, causing a 17-fold increase in circulating levels of HGF as early as 2 hours after PH [3]. Once cleaved and activated, HGF then activates its receptor, Met, within 30–60 minutes after PH [4], initiating a signaling cascade that results in activation of STAT3, PI3K, and Akt [5], [6]. Pre-existing stores of HGF are rapidly depleted and are replaced by HSCs and endothelial cells, which synthesize new HGF [7], [8], [9]. Thus, HGF activation and utilization are crucial events for liver regeneration and provide an early and sustained signal for hepatocyte proliferation [10].

Systemic deletion of HGF causes mid-gestational embryonic lethality due to a defect in placental organogenesis [11]. These mice also have arrested liver development, confirming its essential role in hepatic morphogenesis [12]. Deletion of the HGF receptor Met also results in embryonic lethality, and liver-specific elimination of Met is associated with an impaired or absent regenerative response [13], [14], [15]. However, discrepancies in post-survival surgery make it difficult to determine whether HGF/Met signaling during regeneration functions primarily to facilitate hepatocyte survival or mitogenesis [16]. Previous work in our laboratory has demonstrated lack of suppression of hepatocyte proliferation after in vivo injection of short hairpin RNA sequences against HGF, in view of the fact that there are high concentrations of HGF in the ambient environment of hepatocytes [17]. Therefore, the effect of long-term or chronic suppression of HGF in liver regeneration is unknown.

Recently, Phaneuf et al. generated a mouse with loxP sites flanking exon 5 of the HGF gene (HGF^ex.5 flox^) [18]. No apparent phenotypic differences were observed after recombination, and the proliferative capacity of hepatocytes was only mildly inhibited when these HGF-deleted animals were challenged with carbon tetrachloride (CCl4), a hepatotoxin that causes tissue injury and inflammation resulting in cell death. In order to elucidate the role of HGF in liver homeostasis as well as assess the effect of deleting HGF during surgically-induced liver regeneration, we bred homozygous HGF^ex.5 flox^ mice to mice transgenic for either Mx1-cre or Cre-ER^T^ and then induced cre-mediated recombination. This method allows for rapid and temporal gene inactivation in adult tissues (with Mx1-cre being particularly effective in liver and hematopoietic organs) [19], [20]. We demonstrate that despite genomic recombination and deletion of HGF exon 5, full-length HGF mRNA and protein persist in the liver. However, administration of CCl4 followed by PH depletes this mRNA, causing a diminished regenerative response in mice lacking HGF.

## Materials and Methods

### Ethics Statement

All animal experiments were performed under the guidelines of the National Institutes of Health and the Institutional Animal Use and Care Committee at the University of Pittsburgh. The studies performed in the current report were approved by the Institutional Animal Use and Care Committee at the University of Pittsburgh (protocol #1105844B-4).

### Animals, Induction of Genetic Recombination, CCl4 Treatment, and PH

**Figure 1:**
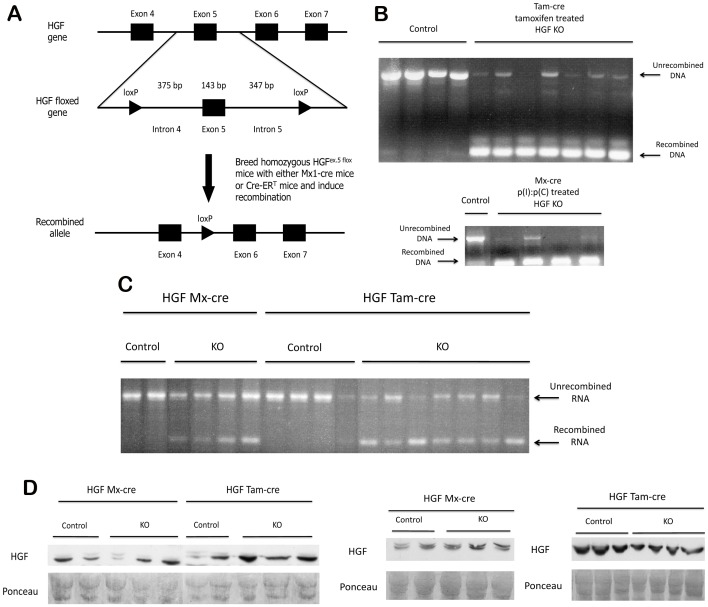
Persistence of unrecombined HGF mRNA and protein in the livers of HGF^ex.5 flox^; Cre^+/−^ mice after genomic recombination. (**A**) Schematic of the targeting strategy for conditional inactivation of the gene for HGF (top). Cre-mediated excision of the floxed HGF allele (middle) leads to the generation of a recombined allele (bottom) lacking exon 5. (**B**) Successful genomic deletion of HGF exon 5 after induction of recombination, as shown by PCR. Top - HGF^ex.5 flox^;Cre-ER^T^ mice; bottom - HGF^ex.5 flox^;Mx1-cre mice. (**C**) RT-PCR shows the presence of both recombined and unrecombined HGF mRNA in KO livers. (**D**) WB for HGF in control and HGF KO livers shows no differences after recombination. Ponceau represents loading control.

A conditional knockout of HGF was generated by breeding homozygous HGF-floxed (HGF^ex.5 flox^) mice (as described previously [18]) to either interferon-inducible Mx1-cre mice or tamoxifen-inducible cre (Cre-ER^T^) mice (all on a C57BL/6 background); the resulting mice had the genotype HGF^ex.5 flox^; Cre^+/−^ (Figure 1A).

Induction of genetic recombination was achieved by intraperitoneal (i.p.) injection of either 100 µl of 10 mg/ml tamoxifen (or corn oil) once daily for 5 days (for the Cre-ER^T^ mice), or 250 µl of 1 mg/ml polyinosinic:polycytidylic ribonucleic acid (pI:pC) (or phosphate buffered saline (PBS)) three times at 2-day intervals (for the Mx1-cre mice). Animals in all conditions were sacrificed beginning at 10 days after the final injection.

For all experiments, homozygous HGF^ex.5 flox^ mice between the ages of 2.5 and 6 months old were used, with equal distribution of males and females in all experimental conditions. Experiments were performed with age- and sex-matched littermates. For more detailed analysis of animal numbers, see Table 1.

**Table 1:** Genotype and number of mice used in experiments.

Basic Characterization				
Genotype	Characterization			
HGF^ex.5 flox^ Mx1-cre Control	4			
HGF^ex.5 flox^ Mx1-cre KO	11			
HGF^ex.5 flox^ Cre-ER^T^ Control	5			
HGF^ex.5 flox^ Cre-ER^T^ KO	10			
**Partial Hepatectomy (PH)**				
**Genotype**	**D2**	**D4**	**D6**	
Control	3	3	3	
HGF KO (Mx-1 cre and Cre-ER^T^)	4	3	3	
**Carbon Tetrachloride (CCl4)**				
**Genotype**	**CCl4**			
HGF^ex.5 flox^ Mx1-cre Control	4			
HGF^ex.5 flox^ Mx1-cre KO	12			
HGF^ex.5 flox^ Cre-ER^T^ Control	4			
HGF^ex.5 flox^ Cre-ER^T^ KO	4			
**PH+CCl4**				
**Genotype**	**D1**	**D2**	**D3**	**D7**
Control	3	5	3	3
HGF KO (Cre-ER^T^ KO)	3	3	4	2

To address the role of HGF in liver regeneration, mice were divided into three experimental groups: partial hepatectomy (PH), carbon tetrachloride (CCl4), and CCl4 followed by PH. In the first group, HGF^ex.5 flox^; Mx1-Cre^+/−^ mice treated with pI:pC and HGF^ex.5 flox^; Cre-ER^T+/−^ mice treated with tamoxifen (as well as vehicle controls for both genotypes)were anesthetized with Isoflurane (Baxter, IL) and subjected to PH by resecting the median and left lateral lobes [4]. In the second group, HGF^ex.5 flox^; Mx1-Cre^+/−^ mice treated with pI:pC and HGF^ex.5 flox^; Cre-ER^T+/−^ mice treated with tamoxifen (as well as vehicle controls for both genotypes) were administered CCl4 i.p. at a dose of 1 µl/g of body weight (which has been shown previously to induce liver regeneration [21]) 1 month after induction of recombination and allowed to recover for at least 1 week before sacrifice. In the third group, HGF^ex.5 flox^; Cre-ER^T+/−^ mice were treated with CCl4≥10 days after a treatment regimen of either tamoxifen or vehicle control, followed by PH 1 month after CCl4. In all groups, livers were harvested for DNA, RNA, and protein as described below.

### Primary Rat Hepatocyte and Non-parenchymal Cell (NPC) Isolation

A single-cell suspension of hepatocytes and NPCs was obtained from homozygous HGF^ex.5 flox^; Cre^+/−^ mice treated 10 days prior with either PBS or p(I):p(C) using a modified calcium two-step collagenase perfusion technique [22]. Hepatocytes were separated from NPCs by low-speed centrifugation (50 *g*, 5 minutes, 4°C). The resulting supernatant was centrifuged at 500 *g* for 10 minutes at 4°C to isolate NPCs. Cells were harvested for DNA, RNA, and protein as described below.

### HSC-T6 Cultures and HGF Treatment

HSC-T6 cells (an immortalized rat liver HSC line [23]) were cultured in Dulbecco’s modified Eagle’s medium (DMEM) containing 10% fetal bovine serum at 37°C in 5% CO_2_ and then treated with varying concentrations of HGF for 48 hours. Cells were harvested for protein and RNA as described below.

### DNA Extraction and Genomic Polymerase Chain Reaction (PCR)

DNA was obtained from the livers of sacrificed mice using phenol/chloroform extraction. PCR was performed in the presence of DMSO and betaine using the following primers, which were designed to detect the wild-type (900 bp), floxed (1200 bp), and recombined (200 bp) forms of HGF genomic DNA:

Forward: 5′ – TGTGACCCTGGATCATCAGTGTAA –3′.

Reverse: 5′ – CGATGTAAATATATGATATGCAAGA –3′.

PCR amplification was carried out as follows: initial denaturation at 94°C for 5 minutes, followed by 35 cycles of denaturation at 94°C for 20 seconds, annealing at 55°C for 30 seconds, extension at 72°C for 1 minute, and 10 minutes of final elongation at 72°C. Samples were run on an agarose gel and visualized using AlphaImager Mini (ProteinSimple, Santa Clara, CA).

### mRNA and RT-PCR

mRNA was isolated and purified from frozen livers using RNA-Bee (Tel-Test, Friendswood, TX). Two µg total RNA per sample was treated with DNase and used for reverse transcription in a 20 µl reaction buffer with random primers and Superscript III (Invitrogen) to generate first-strand cDNA.

PCR was carried out using the Amplitaq Gold kit (Applied Biosystems, Foster City, CA), dNTPs, and the following primers for mouse HGF, which were selected to produce a 266 bp wild-type (unrecombined) product as well as a 123 bp deleted (recombined) product:

Forward: 5′ – CATTGGTAAAGGAGGCAGCTATAAA –3′.

Reverse: 5′ – TTTCACCATTGCAGGTCATGC –3′.

PCR amplification was carried out as follows: initial denaturation at 94°C for 12 minutes, followed by 28 cycles of denaturation at 94°C for 1 minute, annealing at 55°C for 45 seconds, extension at 72°C for 45 seconds, and 10 minutes of final elongation at 72°C. Samples were run on an agarose gel and visualized using AlphaImager Mini (ProteinSimple, Santa Clara, CA).

### Real-time PCR

Quantitative expression levels of unrecombined HGF mRNA were determined by real-time PCR using SYBR green and the following primers for detection of full-length HGF:

Forward: 5′ – CATTGGTAAAGGAGGCAGCTATAAA –3′.

Reverse: 5′ – GGATTTCGACAGTAGTTTTCCTGTAGG –3′.

Reverse-transcribed samples were amplified in parallel on an ABI StepOnePlus instrument (Applied Biosystems). The standard conditions for real-time PCR were as follows: 2 minutes at 50°C, 10 minutes at 95°C followed by 40 cycles of 15 seconds denaturation at 95°C, and elongation at 60°C for 1 minute. Each sample was run in duplicate. Expression levels of HGF were normalized relative to expression of cyclophilin in each sample. Gene expression was calculated by using the 2(−ΔΔCt) method, which was derived from average Ct and expressed as fold change or percent expression of control. Data represent pooled samples from n≥3 animals per genotype per time point, and real-time PCR was repeated at least twice for consistency. Representative assays are shown. For the in vitro HSC-T6 assay, triplicate samples were run in duplicate and average 2(−ΔΔCt) values are shown.

### Protein Extraction, Immunoprecipitation, and Western Blotting

Whole-cell lysates from mouse livers or cell lysates from cultured hepatocytes or NPCs were prepared by homogenization using RIPA buffer (9.1 mmol/L dibasic sodium phosphate, 1.7 mmol/L monobasic sodium phosphate, 150 mmol/L sodium chloride, 1% Nonidet P-40, 0.5% sodium deoxycholate, 0.1% sodium dodecylsulfate [pH adjusted to 7.4]) containing Halt protease inhibitor cocktail (Pierce, Rockford, IL**)**. The concentration of the protein in all lysates was determined by the bicinchoninic acid assay using BSA as a standard.

For immunoprecipitation (IP) studies, 500 µg of cell lysate (prepared in RIPA buffer in the presence of inhibitors) was precleared with mouse IgG together with Protein A/G agarose for 30 minutes at 4°C. After centrifugation, the supernatants were incubated with 20 µg Met antibody (Cell Signaling, Danvers, MA) overnight at 4°C. The next day, samples were incubated with Protein A/G agarose for 1 hour at 4°C. Pellets were collected, washed in PBS containing inhibitors, resuspended in loading buffer, and subjected to electrophoresis, as described below.

Fifty µg of protein from cell or liver lysate or 15–20 µl of eluate from IP studies was subjected to electrophoresis on 7.5% or 4–12% precast sodium dodecyl sulfate polyacrylamide gel electrophoresis (SDS-PAGE) gels using the either the mini-PROTEAN electrophoresis module assembly (Biorad, Hercules, CA) or the Nu-PAGE System (Invitrogen, Carlsbad, CA), followed by transfer to Immobilon-PVDF membranes (Millipore, Bedford, MA). Membranes were stained with Ponceau-S solution to confirm equal loading and then blocked in either 5% nonfat dry milk or 5% BSA in blotto solution (Tris-buffered saline with Tween 20), followed by incubation with primary antibody diluted in 5% milk/blotto or 5% BSA/blotto overnight. Membranes were washed and incubated in horseradish-peroxidase conjugated goat anti-mouse (1∶25,000) and donkey anti-rabbit (1∶40,000) secondary antibodies (Chemicon, Temecula, CA) for 1 hour followed by washing. Proteins were detected by Super-Signal West Pico Chemiluminescent Substrate (Pierce) and visualized by autoradiography. Primary antibodies used in this study were against HGF (1∶500, abcam, Boston, MA), Met (1∶1000) and phosphorylated-Met (Tyr1234/1235; 1∶1000) (Cell Signaling), β-actin (1∶5000, Chemicon), glyceraldehyde 3-phosphate dehydrogenase (GAPDH; 1∶500, Santa Cruz Biotechnology, Santa Cruz, CA) and proliferating cell nuclear antigen (PCNA; 1∶200, Santa Cruz). Blots were stripped with Restore buffer (Pierce) for 10 minutes before re-probing.

### Immunohistochemistry

Immunohistochemistry was performed on mouse liver tissues fixed in 10% formalin, embedded in paraffin, and then sectioned at 4 µm onto Superfrost Plus glass slides. Sections were microwaved in either citrate buffer, ZnSO_4_, or antigen retrieval solution (Dako, Carpinteria, CA), pretreated with 3% H_2_O_2_ to eliminate endogenous peroxidases, and blocked using Ultra V Block (Fisher Scientific, Pittsburgh, PA). Primary antibodies used for this project were against Ki67 (1∶200; Fisher), PCNA (1∶4000; Dako), and HGF (1∶200; abcam). Secondary antibodies were biotinylated donkey anti-mouse and donkey anti-rabbit (Jackson ImmunoResearch Laboratories, Inc., West Grove, PA), both used at a 1∶500 dilution. Immunohistochemistry was performed using the Vectastain ABC Elite kit, developed using DAB (Vector Laboratories, Inc., Burlingame, CA), and counterstained with hematoxylin.

### Statistical Analysis

All experiments were performed three or more times. Representative data from experiments is presented. Autoradiographs of some western blots were scanned and subjected to densitometry using ImageJ software. Statistical assessment for significance was determined using the Student’s *t*-test (two-tailed). A *P* value of less than 0.05 was considered significant (*), and a *P* value of less than 0.01 was considered highly significant (**).

## Results

### Despite Genetic Recombination, Unrecombined HGF mRNA and Protein are Present in the Livers of HGF^ex.5 flox^; Cre^+/−^ Mice

Homozygous HGF^ex.5 flox^; Cre^+/−^ mice treated with a regimen of either tamoxifen or p(I):p(C) to induce global recombination were sacrificed after 10 days. Phenotypically, the livers of recombined animals were indistinguishable from WT livers, with no apparent changes in liver weight, color, or morphology. Further, animals followed for more than 8 months subsequent to deletion of HGF showed no evident phenotype, as assessed by examination of animal behavior and following complete examination of tissues by autopsy. Next, portions of the liver were assessed by PCR to determine the extent of genetic recombination. The presence of a 200 bp band representing the recombined form of the gene was confirmation that exon 5 of HGF had been successfully deleted. Figure 1B shows that in both inducible cre lines, recombination occurred in approximately 70–80% of DNA. To analyze the effect of recombination on HGF mRNA, cDNA was generated from RNA isolated from the livers of these animals. While Figure 1C shows the presence of a truncated HGF mRNA transcript in treated mice, the level of unrecombined HGF mRNA (266 bp) detected in mice that received p(I):p(C) or tamoxifen was not significantly reduced when compared to control animals. Furthermore, analysis of HGF protein from control and p(I):p(C) or tamoxifen treated whole liver lysates by WB showed heterogeneity between animals with no detectable differences in the amount of HGF after recombination (Figure 1D). Thus, in spite of successful genetic recombination of the floxed HGF alleles, a significant amount of full-length unrecombined HGF mRNA and HGF protein persists in the liver.

### Recombination Occurs Equally in Both Hepatocytes and NPCs

**Figure 2:**
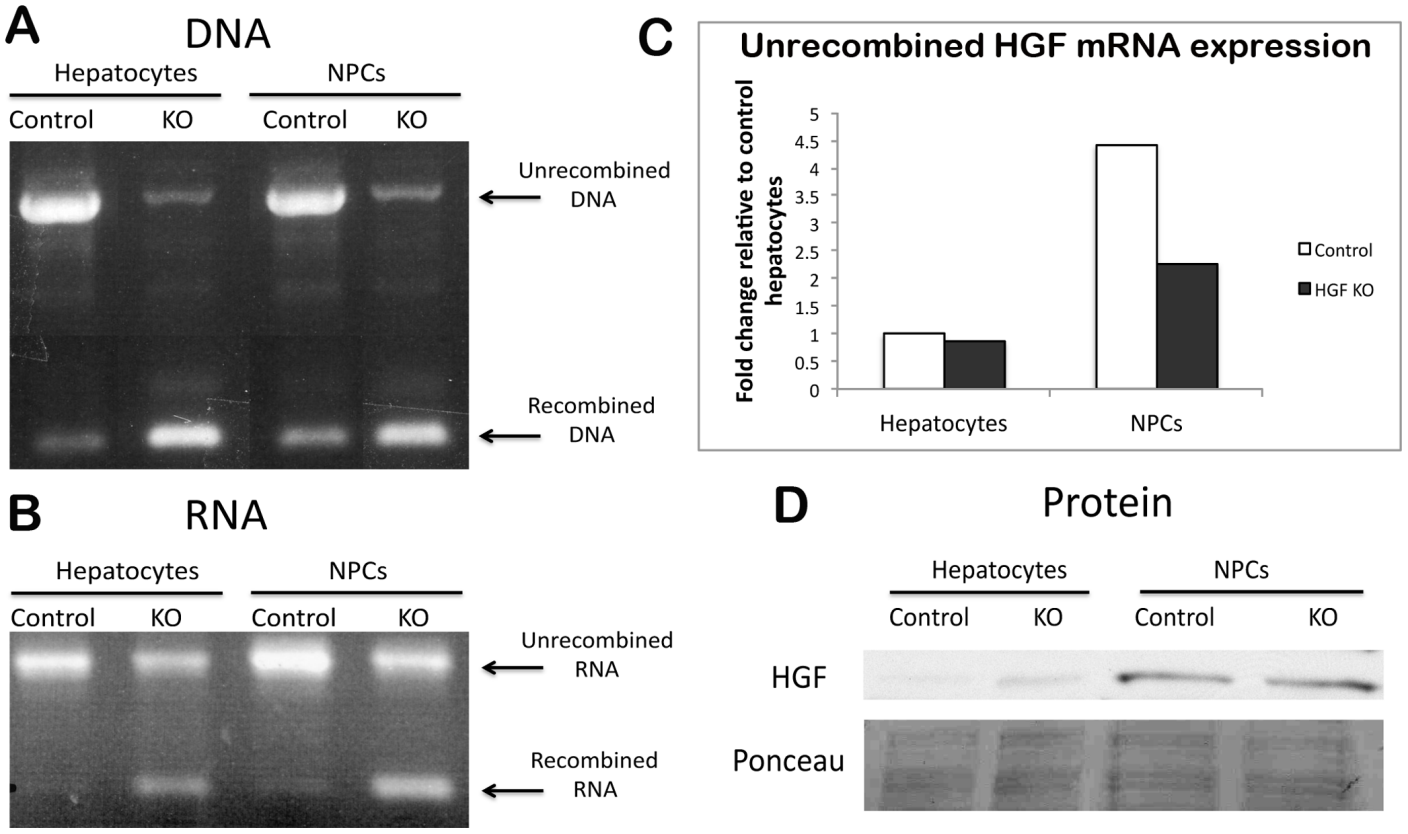
Recombination occurs in all hepatic cell populations, including those that produce HGF. (**A**) Separation of hepatic cell populations from HGF^ex.5 flox^;Mx1-cre mice into hepatocytes and NPCs shows recombination in both after p(I):p(C) treatment as compared to controls. (**B**) RT-PCR shows persistence of unrecombined HGF mRNA in both hepatocytes and NPCs. (**C**) Real-time PCR for full-length HGF mRNA shows a decrease in HGF in the NPC fraction after p(I):p(C) treatment. (**D**) WB for HGF in hepatocytes and NPCs shows that the amount of HGF is unchanged in KOs compared to controls, and is found mainly in the NPC fraction. Ponceau represents loading control.

As HGF is produced by non-parenchymal cells, which comprise a minority subset of the total hepatic cell population [24], [25], we wanted to confirm that treatment with agents that induce recombination targets all liver cell populations non-preferentially. Because Mx1-cre and Cre-ER^T^ were indistinguishable in their ability to cause recombination of the HGF gene in the initial characterization, we chose to utilize Mx1-cre mice for the cell separation experiments. We isolated hepatocytes and NPC fractions from PBS- and p(I):p(C)-treated HGF^ex.5 flox^; Mx1-cre mice and processed the cells for DNA, RNA, and protein analysis as above. Successful genetic recombination was evident in the cell fractions of both hepatocytes and NPCs as compared to controls (Figure 2A). Analysis of HGF mRNA showed the presence of recombined HGF mRNA in both hepatocyte and NPC fractions from p(I):p(C)-treated livers, with NPCs expressing over four-fold higher levels of HGF than hepatocytes before recombination; this expression was reduced by half after deletion of HGF exon 5 (Figure 2B, C). However, as in whole liver, the presence of unrecombined HGF mRNA is still detectable in both fractions. The presence of HGF mRNA in the “hepatocyte” fraction reflects the standard contamination of such hepatocyte isolates by 3–5% with non-parenchymal cells. The amount of HGF protein remained unchanged after p(I):p(C) treatment in both hepatocytes and NPCs, with HGF expression being primarily localized to the NPC fraction (Figure 2D). Thus, genetic recombination occurs not only in hepatocytes, but also in the fraction of hepatic cells that produce HGF.

### PH as a Stand-alone Event Decreases the Amount of Unrecombined HGF mRNA in Liver; however, HGF Protein and Proliferation are Unaffected

**Figure 3:**
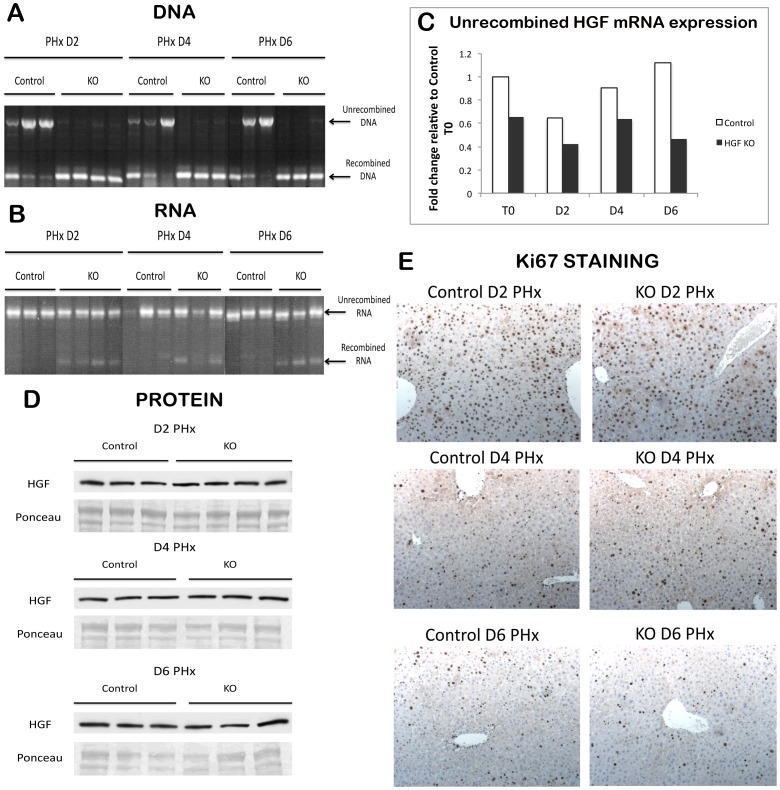
No change in amount of HGF protein or hepatocyte proliferation in HGF KO mice after PH. (**A**) Genomic recombination is present in cre-inducible HGF^ex.5 flox^ KO mice at all time points after PH, as assessed by PCR. (**B**) RT-PCR shows a significant amount of full-length HGF mRNA remaining in KOs even after PH. (**C**) The amount of unrecombined HGF is slightly decreased in HGF KO as compared to controls before and after PH, as assessed by real-time PCR. (**D**) Comparable amounts of HGF protein in control and HGF KO livers after PH. Ponceau represents loading control. (**E**) Proliferation is unaffected in HGF KO mice after PH, as shown by representative images of Ki67 IHC (100X).

Despite lack of impact on mRNA and protein, we proceeded to determine whether genetic deletion of HGF would adversely affect liver regeneration, and performed PH on HGF^ex.5 flox^ Mx1-cre or Cre-ER^T^ mice treated to induce recombination (hereafter referred to as HGF KO mice) as well as control HGF^ex.5 flox^ untreated animals. Livers were harvested at days 2, 4, and 6 after PH. In mouse livers prior to PH, there was a near-complete conversion of the floxed HGF allele to the recombined fragment in KO mice as compared to controls (Figure 3A). However, mRNA analysis shows that the full-length form of HGF persists even after PH (Figure 3B). In order to detect subtle changes in HGF mRNA expression that may not be apparent in RT-PCR, we also performed quantitative real-time PCR, which showed a blunted expression of full-length HGF as compared to controls, especially at D6 (Figure 3C). Interestingly, despite a decrease in unrecombined HGF mRNA, HGF protein expression in KO livers is equivalent to controls at all time points analyzed (Figure 3D). Finally, Ki67, which identifies cells in the S-phase of the cell cycle, is also equivalent in both control and HGF KO after PH at all time points analyzed (Figure 3E). Thus, despite a depletion of functional HGF mRNA, liver regeneration is unaffected in HGF KO mice after PH, consistent with persistent HGF protein.

### Production of HGF by HSCs is Regulated by the Concentration of HGF in Culture

**Figure 4:**
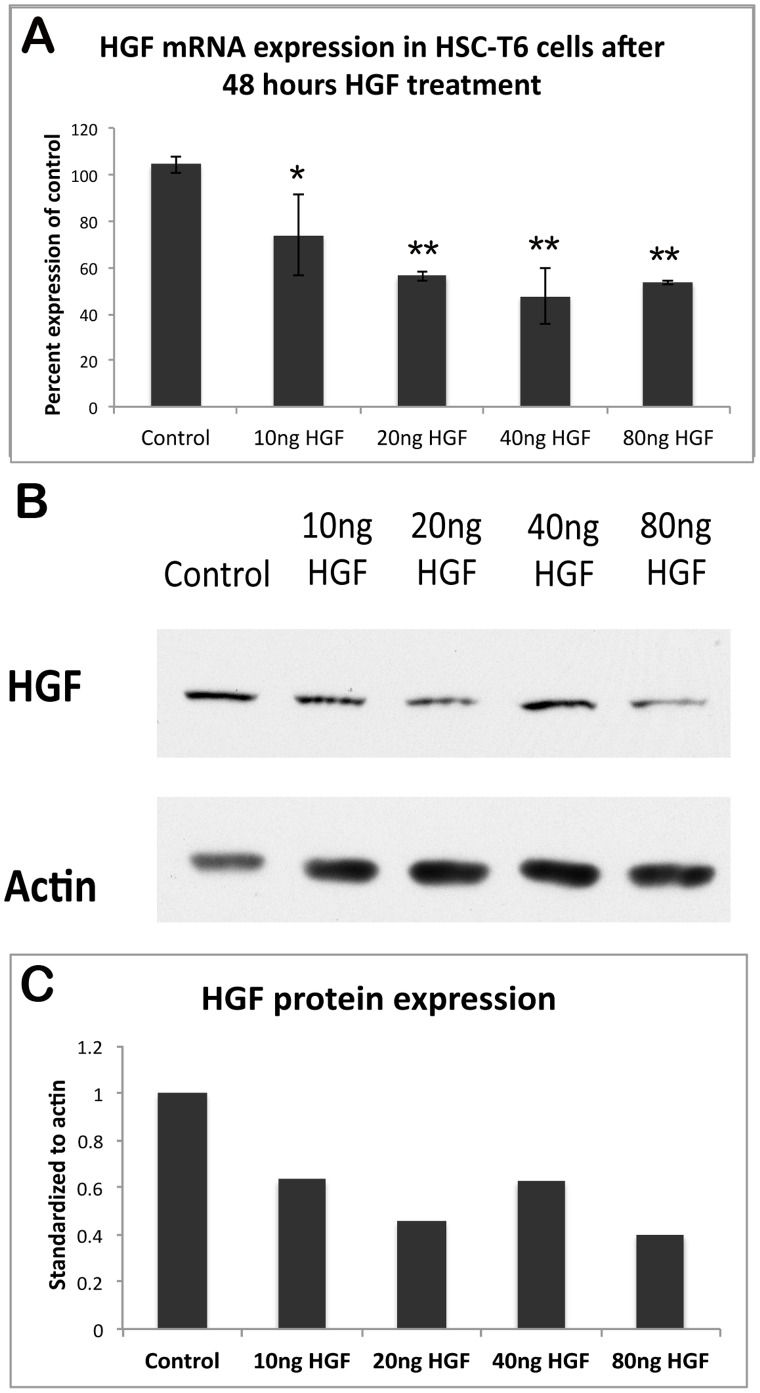
Inverse correlation between the concentration of HGF in culture and production of HGF by HSCs. (**A**) Real-time PCR for HGF mRNA shows a dose-dependent decrease in HGF production by HSC-T6 cells in response to increasing concentrations of HGF in culture (*P<0.05; **P<0.01). (**B**) HGF protein production by HSC-T6 cells decreases in response to increased HGF, as measured by WB. Actin represents loading control. (**C**) Densitometry analysis on representative WB shown in (B).

To determine if the population of HSCs containing unrecombined genomic DNA can compensate for an environmental deficit of HGF by increasing de novo production of full-length HGF, we tested the reverse - whether HSCs in culture could suppress production in response to an increase in HGF. HSC-T6 cells actively suppress HGF mRNA in a dose-dependent manner in response to concentrations of HGF as low as 10 ng/ml, as shown in Figure 4A. HGF protein is also decreased upon addition of HGF to the media (Figure 4B, C). Thus, HSCs are able to regulate HGF production by sensing homeostatic perturbations in ambient HGF and responding accordingly.

### HGF mRNA and Protein are Significantly Decreased in HGF^ex.5 flox^; Cre^+/−^ Mice Treated with CCl4 after Recombination

**Figure 5:**
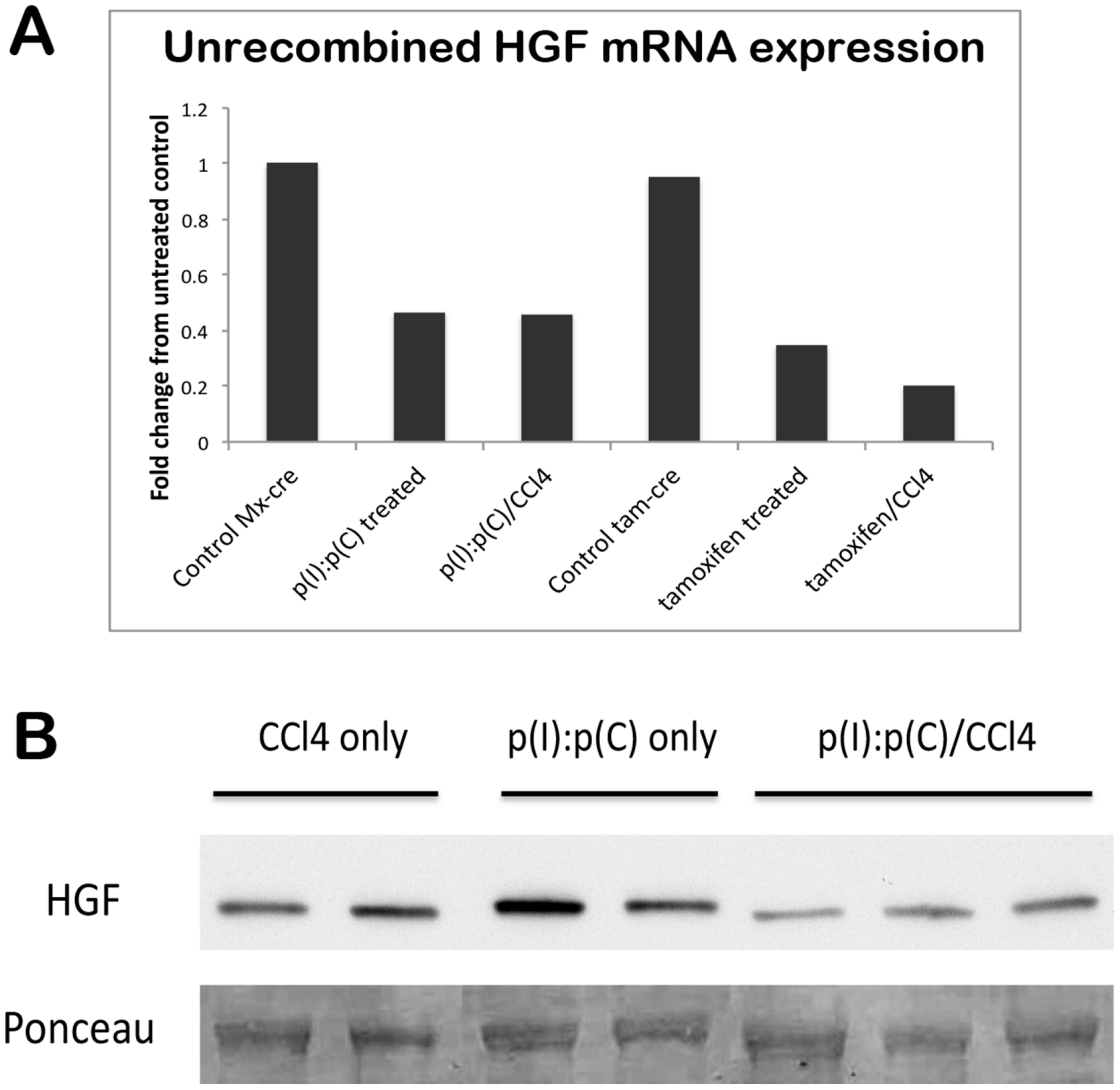
Liver regeneration stimulated by CCl4 depletes HGF mRNA and protein in HGF KO mice. (**A**) CCl4 treatment following genomic recombination further decreases full-length HGF mRNA, as assessed by real-time PCR. (**B**) WB shows decreased HGF expression in livers of HGF KO mice treated with CCl4 in combination with p(I):p(C), as compared to controls or those treated with p(I):p(C) only. Ponceau represents loading control.

In view of the results shown in Figure 3B, in an attempt to deplete the persisting unrecombined HGF mRNA, HGF KO mice were subjected to CCl4, a hepatotoxin that induces liver damage and regeneration. Figure 5A shows that HGF^ex.5 flox^; Cre^+/−^ mice treated with either p(I):p(C) or tamoxifen to induce recombination show a greater than 50% decrease in unrecombined HGF mRNA; however, when these mice were stimulated into liver regeneration by treatment with CCl4 at 1 month after the cessation of the cre-inducing protocol, HGF mRNA expression decreases even further in HGF^ex.5 flox^; Cre-ER^T^ KO livers, to 20% that of controls. HGF^ex.5 flox^; Mx1-cre livers also show a significant and measurable decrease in HGF protein expression, as demonstrated by WB (Figure 5B). Thus, induction of liver regeneration by CCl4 forces the HGF KO liver to deplete existing stores of unrecombined HGF mRNA and protein, mandating de novo HGF synthesis in the liver upon future insults or surgical manipulations.

### CCl4 and PH in HGF^ex.5 flox^; Cre-ER^T^ KO Mice Results in Compromised Liver Regeneration Due to a Decrease in HGF

**Figure 6:**
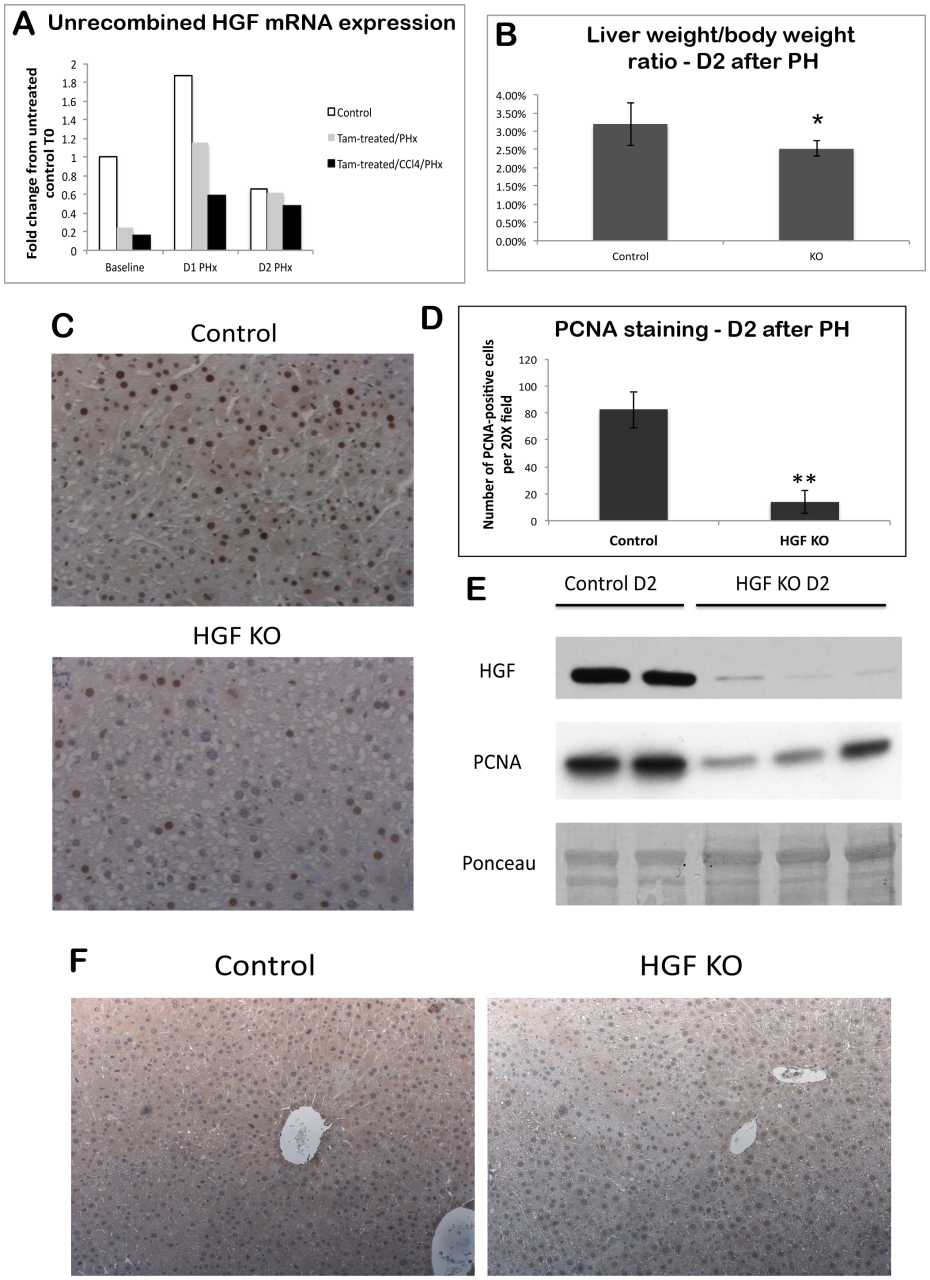
Liver regeneration is compromised in HGF KO mice after CCl4 and PH due to a decrease in HGF. (**A**) Real-time PCR demonstrates decreased HGF mRNA in HGF^ex.5 flox^; Cre-ER^T^ KO mice treated with both CCl4 and PH as compared to control at D1 after PH. (**B**) Graph of liver weight to body weight ratios after CCl4/PH shows a significant decrease in HGF KOs at D2. (*P<0.05) (**C**) Representative images of PCNA IHC on livers harvested at D2 after PH in control and HGF KO animals treated with CCl4 (200X) (**D**) Quantification of PCNA staining shown in (C). A total of 5 fields per liver (n = 3 per condition) were counted. (**P<0.01) (**E**) WB for HGF and PCNA shows a dramatic decrease in both proteins in HGF KO animals at D2 after CCl4/PH. Ponceau represents loading control. (**F**) HGF IHC in control and HGF KO livers harvested at D2 after PH demonstrates sparse and irregular distribution of HGF protein.

Finally, we performed PH on control and HGF^ex.5 flox^; Cre-ER^T^ KO mice after they had recovered and fully regenerated from the administration of CCl4, in order to induce de novo synthesis of truncated, non-functional HGF in KO and thus assess the impact on liver regeneration. HGF KO mice triggered into liver regeneration first by CCl4 and (after 1 month) followed by PH have a 3-fold reduction in HGF mRNA at D1, as compared to KO mice given PH only, which have a 2-fold decrease (Figure 6A). Interestingly, the amount of HGF mRNA at D2 in KOs is almost equivalent to that of controls, suggesting a persistent compensatory or feedback mechanism. The liver weight to body weight (lw/bw) ratio in HGF KO mice was significantly less at D2 after PH (Figure 6B), indicating a defect in regeneration that could result from a lack of HGF evident at D1. These findings coincided with fewer numbers of proliferating hepatocytes in HGF KO mice at D2, as measured by PCNA staining (Figure 6C, D, E). However, proliferation at D3 and D7 is comparable to WT (data not shown), and all KO mice survive surgery, indicating that deletion of HGF delays, but does not abolish, liver regeneration.

**Figure 7:**
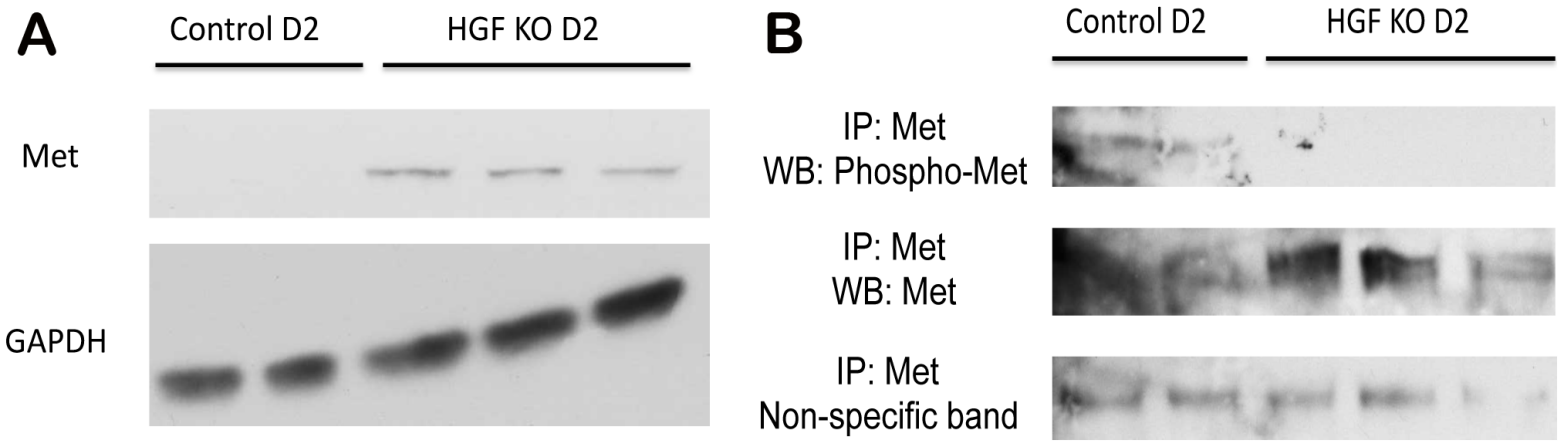
Met expression and activation is altered in HGF KO mice after CCl4 and PH. (**A**) WB for total Met shows increased expression in HGF KO animals at D2 after CCl4/PH. GAPDH represents loading control. (**B**) IP shows that Met is phosphorylated and active in controls but not in HGF KOs at D2 after CCl4/PH. Successful pulldown of Met is verified, and a non-specific band is used as normalizing control.

Analysis of HGF by WB demonstrates a noteworthy decrease in HGF protein in KOs at D2 as compared to controls (Figure 6E), which is confirmed by IHC for HGF, which shows sparse and irregular distribution of HGF expression (Figure 6F). This occurs despite some persistence of the unrecombined form of HGF mRNA shown in Fig. 6A. Intriguingly, levels of the HGF receptor Met were higher in HGF KO livers at D2 after PH (Figure 7A). However, there was a dramatic decrease in the tyrosine-phosphorylated (active) form of Met in HGF KOs as assessed by IP for Met, suggesting that signaling through Met is impaired in the absence of HGF (Fig. 7B). Thus, depletion of unrecombined HGF mRNA below a certain level by induction of two sequential cycles of regeneration through CCl4 and PH finally resulted in delayed liver regeneration in HGF KO mice.

## Discussion

HGF has been implicated as an initiator of liver regeneration because it is a direct mitogen for hepatocytes in culture, it is the only hepatocyte mitogen detectable in the plasma after PH, it activates signal transduction very early after PH, and it can cause massive hepatic enlargement and hepatocyte DNA synthesis when injected in mice and rats [4], [22], [26], [27], [28], [29]. As genetic deletion of HGF or Met is embryonic lethal, it has thus far not been possible to study the contribution of HGF/Met signaling to liver regeneration in the sustained absence of this signaling pathway. Therefore, we employed the cre-loxP system to ablate HGF in adult mice.

Although HSCs are the primary source of HGF during liver regeneration [9], there are two major hurdles to utilizing a stellate cell-specific knockout. First, the HSC population in the liver is known to be heterogeneous [30], [31], and thus one marker alone is insufficient to isolate or characterize these cells. Therefore, cre-mediated deletion using a single stellate cell-specific promoter would only delete the floxed gene from a subset of stellate cells. Second, other cell types, such as endothelial cells, are also known to express HGF in the liver, particularly during liver regeneration [8], [32]. The rationale behind utilizing the global conditional deletion is to remove HGF from all cell types in the liver in a non-biased way.

HGF consists of an α-subunit containing four kringle domains and a β-subunit pseudo-protease domain [33]. Mutations of HGF lacking the first or second kringle domains are unable to bind to and activate the Met receptor [34]. Therefore, we utilized a mouse model in which exon 5 of the HGF gene, which encodes most of the first kringle domain, was deleted [18]. We demonstrated that HGF is an essential component of an efficient regenerative response after PH, and in the absence of this growth factor, liver regeneration is delayed. However, the results were complicated by the fact that full-length HGF mRNA and protein were present in the liver despite efficient recombination of the HGF gene, and treatment with two sequential regenerative stimuli (CCl4 followed by PH) was necessary to sufficiently deplete the stores of unrecombined HGF and create a dependency on de novo synthesis.

The persistence of full-length HGF mRNA in the liver suggests two possibilities, either of which may occur: first, that HGF mRNA is long-lived; and second, that HSCs with unrecombined mRNA can regulate production of HGF in response to fluctuations in the amount of HGF present in their environment and maintain sufficient amounts of HGF mRNA and protein. The half-life of HGF mRNA has been reported in the literature to be on the order of minutes to hours rather than days [35], [36], [37]. However, these measurements were performed in actively proliferating cells, whereas in normal resting liver, stellate cells (and hepatocytes, perhaps the most important cell population recipient of HGF protein) are quiescent and thus would have less need for rapid turnover of growth factor mRNAs. It is known that the half-lives of many mRNAs are determined by *trans*-acting factors, such as hormones, growth factors, ions, and cytokines, which can influence mRNA longevity by protecting it from degradation [38]. Furthermore, mRNA half-life is closely tied to its physiological function [39], and since HGF is stored in an inactive form in the liver extracellular matrix (ECM), it is conceivable that HGF mRNA turnover in normal liver is low. In support of the argument for a long half-life for HGF mRNA is the fact that the amount (or percent of total) of intact unrecombined HGF mRNA was decreased by two sequential regenerative episodes. However, we also found that the stellate cell line HSC-T6 was able to decrease production of HGF in response to increasing concentrations of HGF in media. Others have also shown that HSCs express Met and can thus respond to HGF [40], [41]. Therefore, a small number of stellate cells with unrecombined mRNA may be able to respond to low levels of HGF and compensate by producing more HGF protein. Both of the above mechanisms may contribute to the preservation of HGF mRNA in KO mice after recombination.

One episode of liver regeneration alone causes a decrease in residual unrecombined HGF mRNA (Figure 3). This is because liver regeneration activates synthesis of new HGF, and active translation of any mRNA typically results in a portion of the translated mRNA being degraded. Thus, we wanted to use two consecutive rounds of regeneration – the first to deplete existing HGF, and the second to force transcription of HGF – in order to determine the effect of HGF loss on liver regeneration. In view of operative difficulties in performing two sequential partial hepatectomies, we used pretreatment with CCl4 as the first regenerative event to ensure pre-existing stores of HGF are consumed by CCl4 prior to PH. This would thus create truncated, inactive HGF protein in the KO during the next cycle of liver regeneration (PH), as an increasingly larger percentage of HGF would have to be derived from the truncated mRNA. CCl4-induced liver regeneration is not as standardized as that induced by PH due to confounding factors such as inflammation, injury, and necrosis. However, a previous study has shown that plasma HGF levels are prolonged after CCl4 due to enhanced synthesis of HGF, and thus overall CCl4-mediated liver regeneration requires more HGF than normal liver regeneration [3]. Indeed, we show that pretreatment with CCl4 rapidly depletes the unrecombined preexisting HGF (Figure 5). Our study indicates that inactive HGF stored in the matrix may be enough to drive hepatocyte proliferation in HGF KO mice without the need for de novo synthesis, and only after preexisting stores of HGF are utilized and depleted does deficient production of HGF compromise liver regeneration.

Another intriguing observation was the significant increase in Met receptor expression in HGF KOs 2 days after PH. Phosphorylation and activation of Met is known to lead to ubiquitination and subsequent degradation [42], which may explain the absence of Met in WT livers at D2. Indeed, we found evident phosphorylation of Met in WTs, while Met activation in HGF KOs was eliminated, as demonstrated by detection of the tyrosine-phosphorylated form of Met by IP. Thus, continued expression of Met in KO may reflect the lack of ongoing HGF needed to activate this signaling pathway, preventing Met degradation which occurs after HGF ligation with its receptor [42].

Inducible inactivation of floxed alleles is an interesting approach to study the essentiality of growth factors. This is especially true in the case of HGF/Met signaling, since there is only one receptor for HGF (i.e. c-Met) and only one ligand for c-Met (i.e. HGF); hence, the unique one-on-one relationship between these cognate partners is the only one which is capable of causing uncompensated signaling loss if it were to fail. Our studies, however, demonstrate the caution required to fully interpret findings with deletion of growth factors. In the case of HGF, systemic deletion is embryonic lethal. The phenotypes caused by systemic deletion of either HGF or Met are identical. Contrary to this, we find that postnatal deletion of HGF is not associated with any phenotype. Careful analysis, however, demonstrates that the presence of HGF, though difficult to eliminate by simple standard genetic manipulation, is important during liver regeneration. This is demonstrable only after all efforts have been taken to decrease HGF to very low levels in the ambient environment of the hepatocytes, and in the absence of these efforts, erroneous conclusions could be drawn. Overall, the HGF KO mouse gave us the opportunity to gain further insight into the complexities of the role and regulation of HGF expression during liver regeneration.
