# Correction: Conditional Genetic Elimination of Hepatocyte Growth Factor in Mice Compromises Liver Regeneration after Partial Hepatectomy

**DOI:** 10.1371/journal.pone.0282358

**Published:** 2023-02-23

**Authors:** Kari Nejak-Bowen, Anne Orr, William C. Bowen, George K. Michalopoulos

After this article [1] was published, concerns were raised about horizontal and vertical discontinuities in the western blots in Figs 1, 2, 3 and 5 in article [1]. Specifically:

- In the top panel in Fig 1B there are vertical discontinuities between lanes 3 and 4, 4 and 5, 6 and 7, and 10 and 11.
- In the bottom panel in Fig 1B there are vertical discontinuities between lanes 1 and 2, and 2 and 3.
- In Fig 1C there are vertical discontinuities between lanes 1 and 2, 2 and 3, 3 and 4, 9 and 10, 10 and 11, 12 and 13, and 16 and 17.
- In both the HGF and Ponceau blots in the far right, HGF Tam-cre, panels in Fig 1D there are vertical discontinuities between lanes 3 and 4.
- In Fig 2A there:
  ○ Are vertical discontinuities between lanes 1 and 2, 2 and 3, and 3 and 4.
  ○ Is a horizontal discontinuity approximately two thirds of the way down across all lanes.
- In both Fig 3A and 3B there are vertical discontinuities between lanes 1 and 2, 3 and 4, 5 and 6, 7 and 8, 8 and 9, 10 and 11, 11 and 12, 13 and 14, 14 and 15, 16 and 17, and 18 and 19.
- In both the HGF and Ponceau blots in Fig 5B there are vertical discontinuities between lanes 2 and 3, and 4 and 5.

In editorial follow up on these issues, images and individual-level quantitative data belonging to the original experiments were provided by the corresponding author for all figures of concern (S1 File). The corresponding author stated that the discontinuities in the blots are a result of rearranging parts of the original images in order to group together similar gel items such as controls, knockouts and timepoints. The corresponding author also noted that in all panels in Fig 3D, the original labels showing controls on the left and KO samples on the right are incorrect. The correct version of Fig 3D with alternating control and KO labels is provided here and the original underlying data for Fig 3D are in S1 File. Updated versions of Figs 1B, 1C, 1D, 2A, 3A, 3B and 5B are also provided here where the lanes, and corresponding labels, have the same order as the original underlying blots and gels in S1 File. For Fig 2A, the horizontal discontinuity has also been removed in the updated version below. The authors stated that the presentation of the corrected figures does not affect the conclusions.

**Fig 1:**
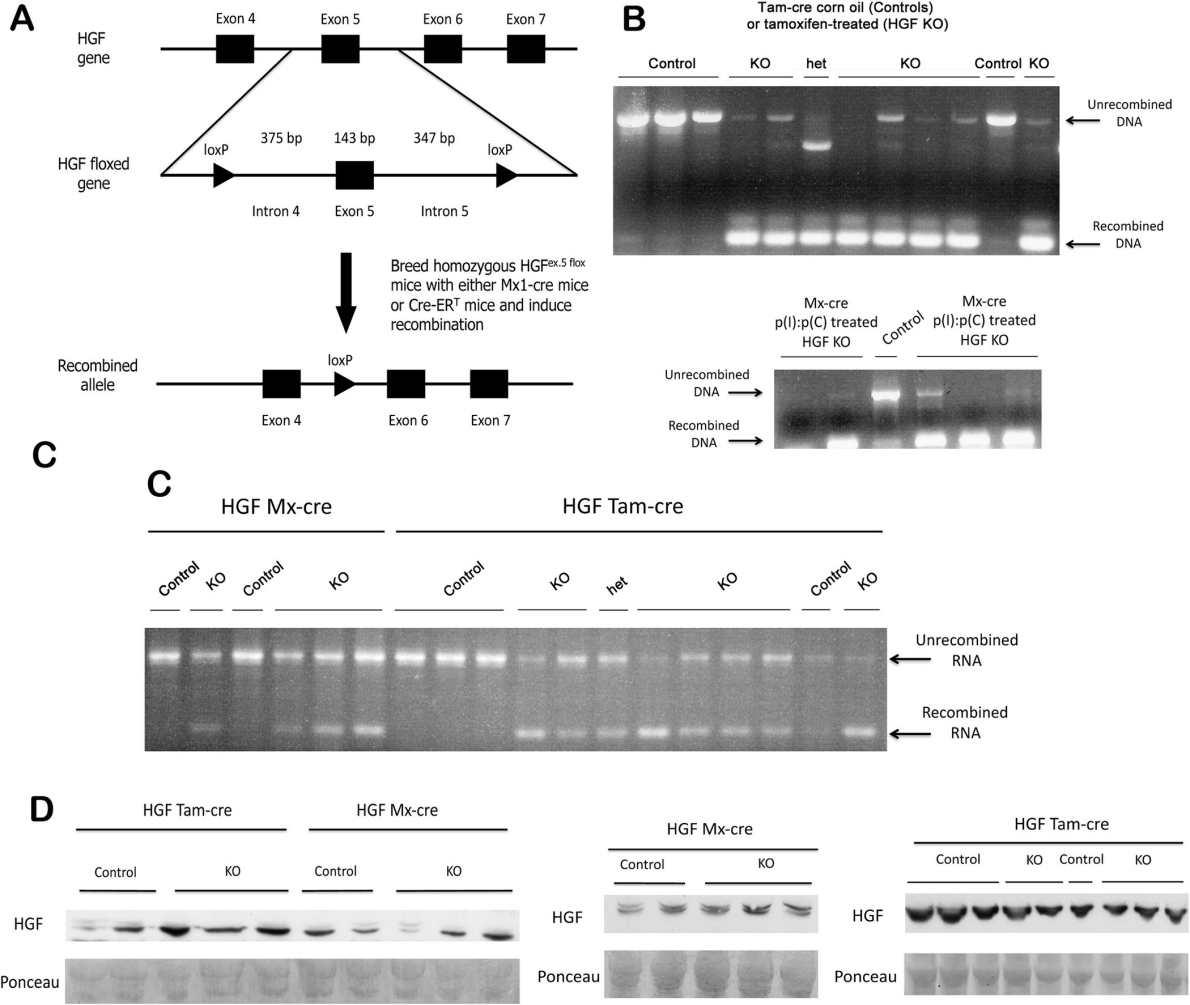
Persistence of unrecombined HGF mRNA and protein in the livers of HGF^ex.5 flox^; Cre^+/-^ mice after genomic recombination. (**A**) Schematic of the targeting strategy for conditional inactivation of the gene for HGF (top). Cre-mediated excision of the floxed HGF allele (middle) leads to the generation of a recombined allele (bottom) lacking exon 5. (**B**) Successful genomic deletion of HGF exon 5 after induction of recombination, as shown by PCR. Top—HGF^ex.5 flox^;Cre-ER^T^ mice; bottom—HGF^ex.5 flox^;Mx1-cre mice. (**C**) RT-PCR shows the presence of both recombined and unrecombined HGF mRNA in KO livers. (**D**) WB for HGF in control and HGF KO livers shows no differences after recombination. Ponceau represents loading control.

**Fig 2:**
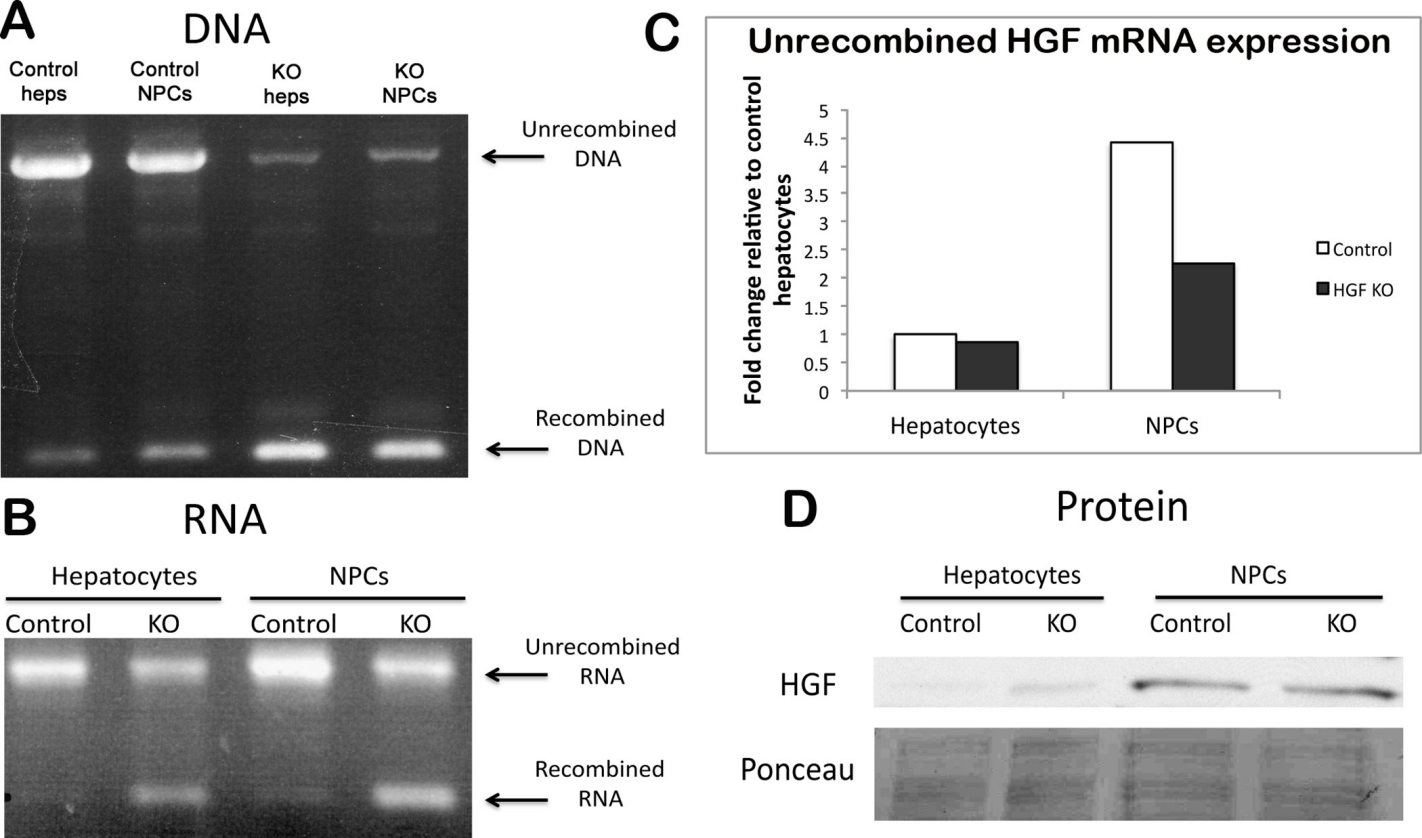
Recombination occurs in all hepatic cell populations, including those that produce HGF. (**A**) Separation of hepatic cell populations from HGF^ex.5 flox^;Mx1-cre mice into hepatocytes and NPCs shows recombination in both after p(I):p(C) treatment as compared to controls. (**B**) RT-PCR shows persistence of unrecombined HGF mRNA in both hepatocytes and NPCs. (**C**) Real-time PCR for full-length HGF mRNA shows a decrease in HGF in the NPC fraction after p(I):p(C) treatment. (**D**) WB for HGF in hepatocytes and NPCs shows that the amount of HGF is unchanged in KOs compared to controls, and is found mainly in the NPC fraction. Ponceau represents loading control.

**Fig 3:**
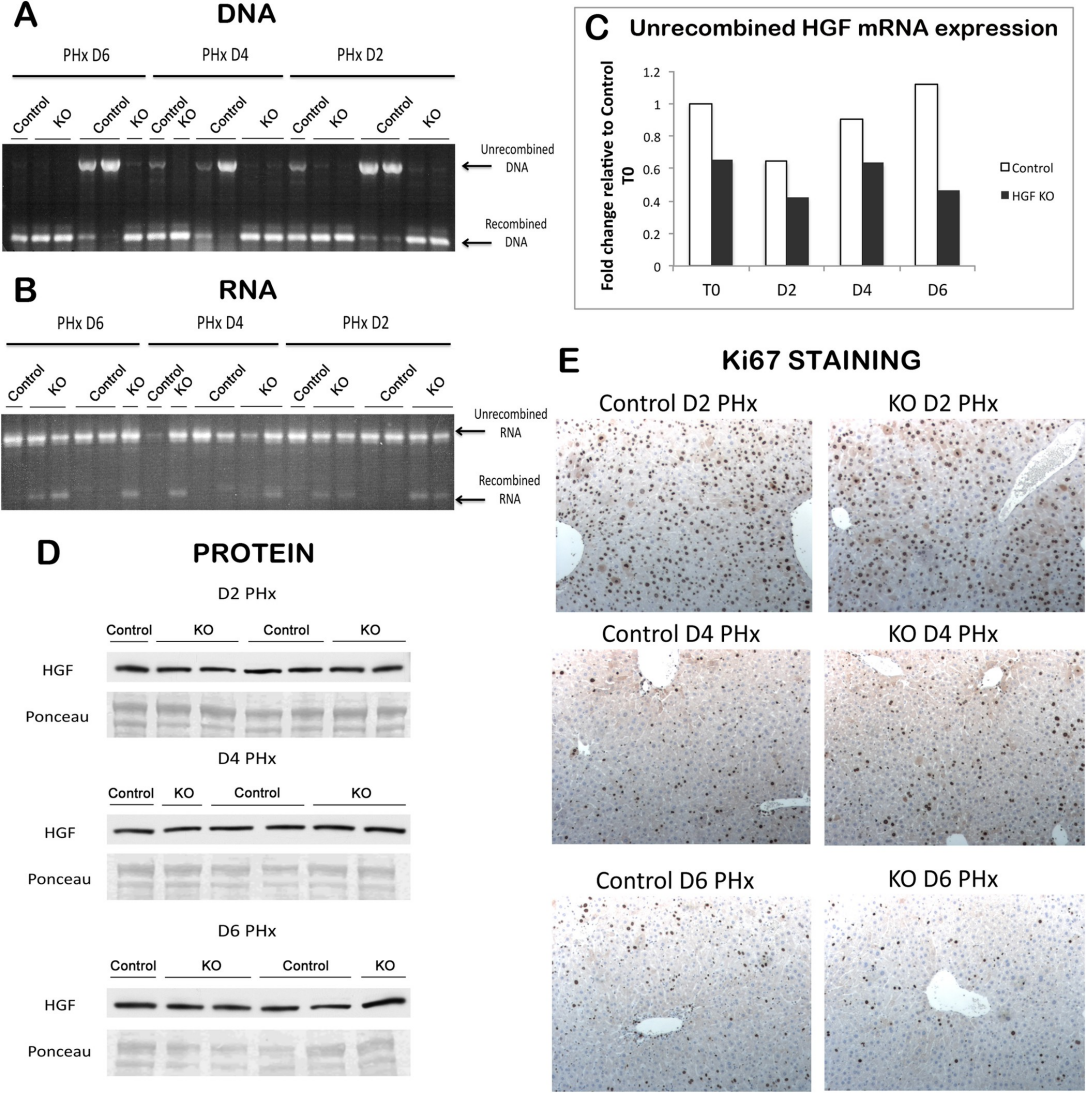
No change in amount of HGF protein or hepatocyte proliferation in HGF KO mice after PH. (**A**) Genomic recombination is present in cre-inducible HGF^ex.5 flox^ KO mice at all time points after PH, as assessed by PCR. (**B**) RT-PCR shows a significant amount of full-length HGF mRNA remaining in KOs even after PH. (**C**) The amount of unrecombined HGF is slightly decreased in HGF KO as compared to controls before and after PH, as assessed by real-time PCR. (**D**) Comparable amounts of HGF protein in control and HGF KO livers after PH. Ponceau represents loading control. (**E**) Proliferation is unaffected in HGF KO mice after PH, as shown by representative images of Ki67 IHC (100X).

**Fig 5:**
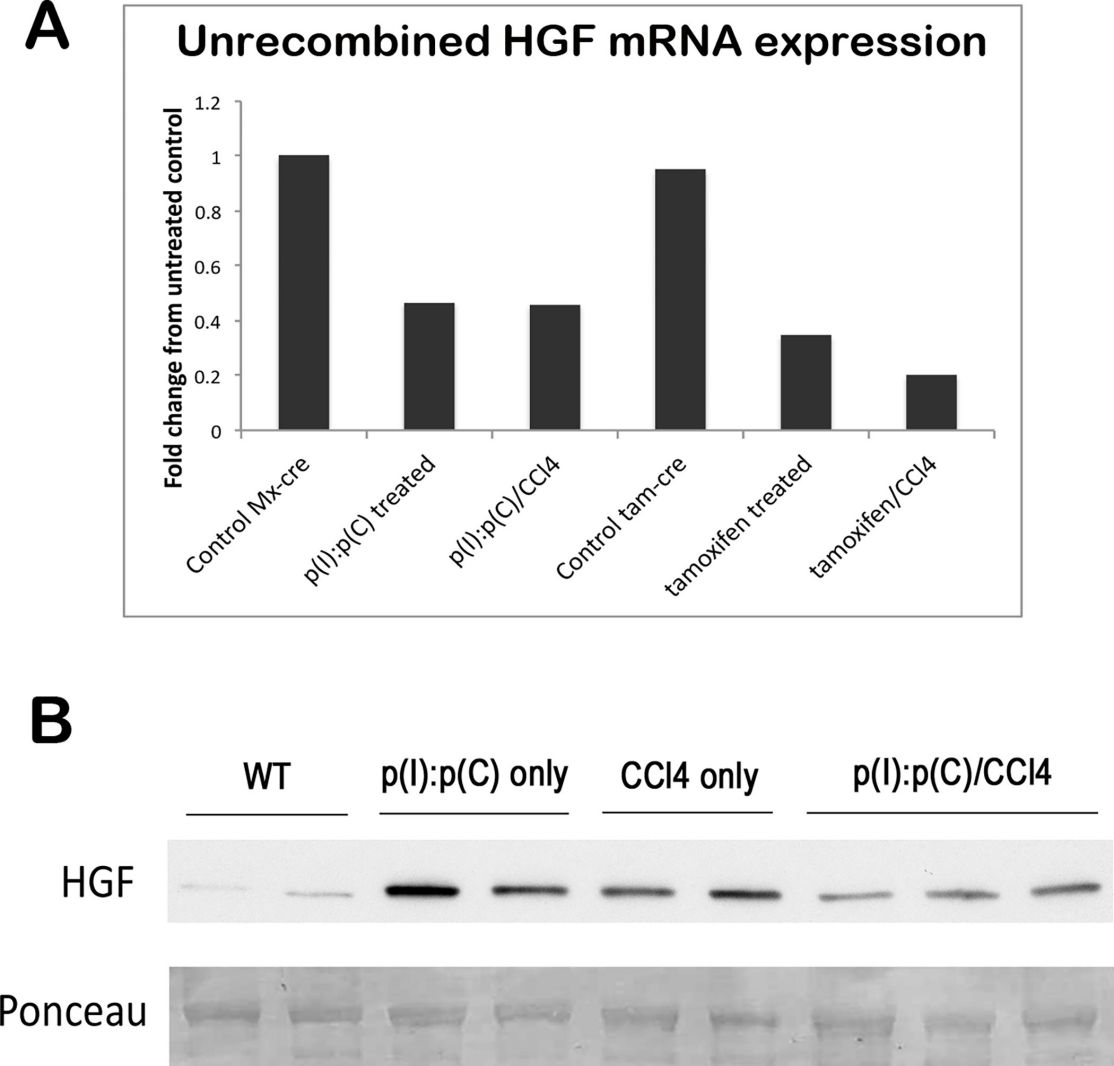
Liver regeneration stimulated by CCl4 depletes HGF mRNA and protein in HGF KO mice. (**A**) CCl4 treatment following genomic recombination further decreases full-length HGF mRNA, as assessed by real-time PCR. (**B**) WB shows decreased HGF expression in livers of HGF KO mice treated with CCl4 in combination with p(I):p(C), as compared to controls or those treated with p(I):p(C) only. Ponceau represents loading control.

## Supporting information

S1 File Underlying data supporting Figs 1–7. (ZIP)
