# Supplementary figures and images for: Correction: Conditional Genetic Elimination of Hepatocyte Growth Factor in Mice Compromises Liver Regeneration after Partial Hepatectomy
Source: PLoS One. 2023 Feb 23;18(2):e0282358. doi: 10.1371/journal.pone.0282358 (PMC9949624; doi:10.1371/journal.pone.0282358)

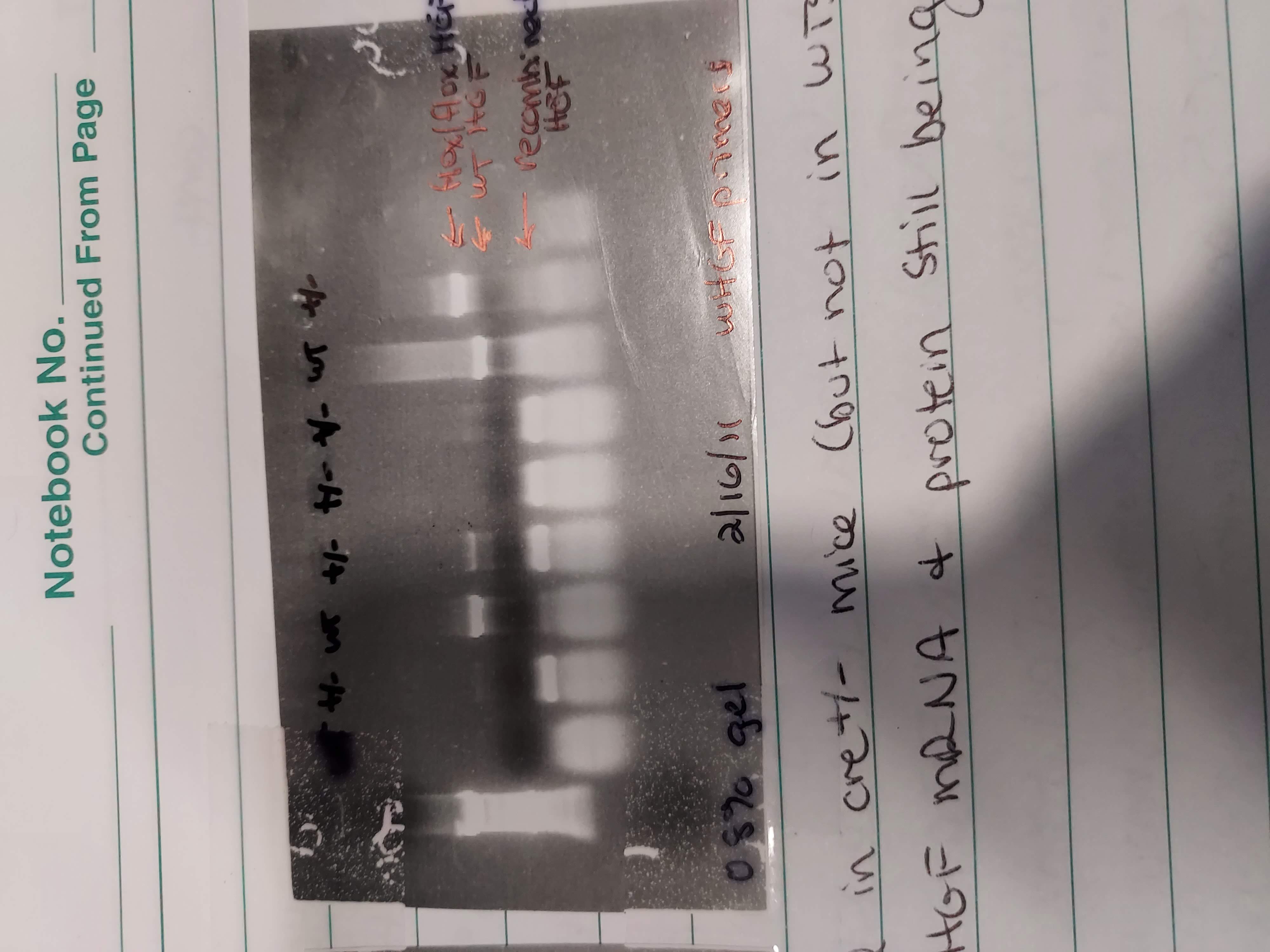

Supplement: S1 File — (ZIP) [file pone.0282358.s001.zip › PLOS ONE images/Fig1Bbottom-1.jpg]

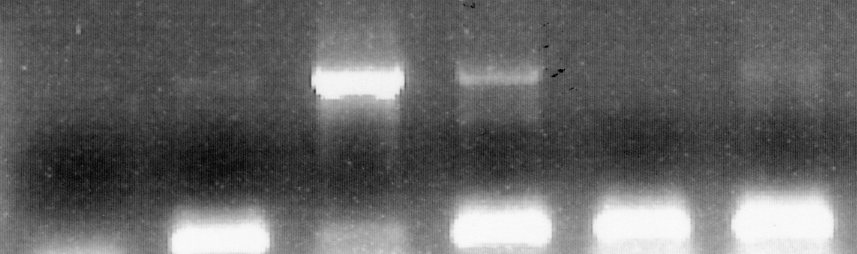

Supplement: S1 File — (ZIP) [file pone.0282358.s001.zip › PLOS ONE images/Fig1Bbottom-2.jpg]

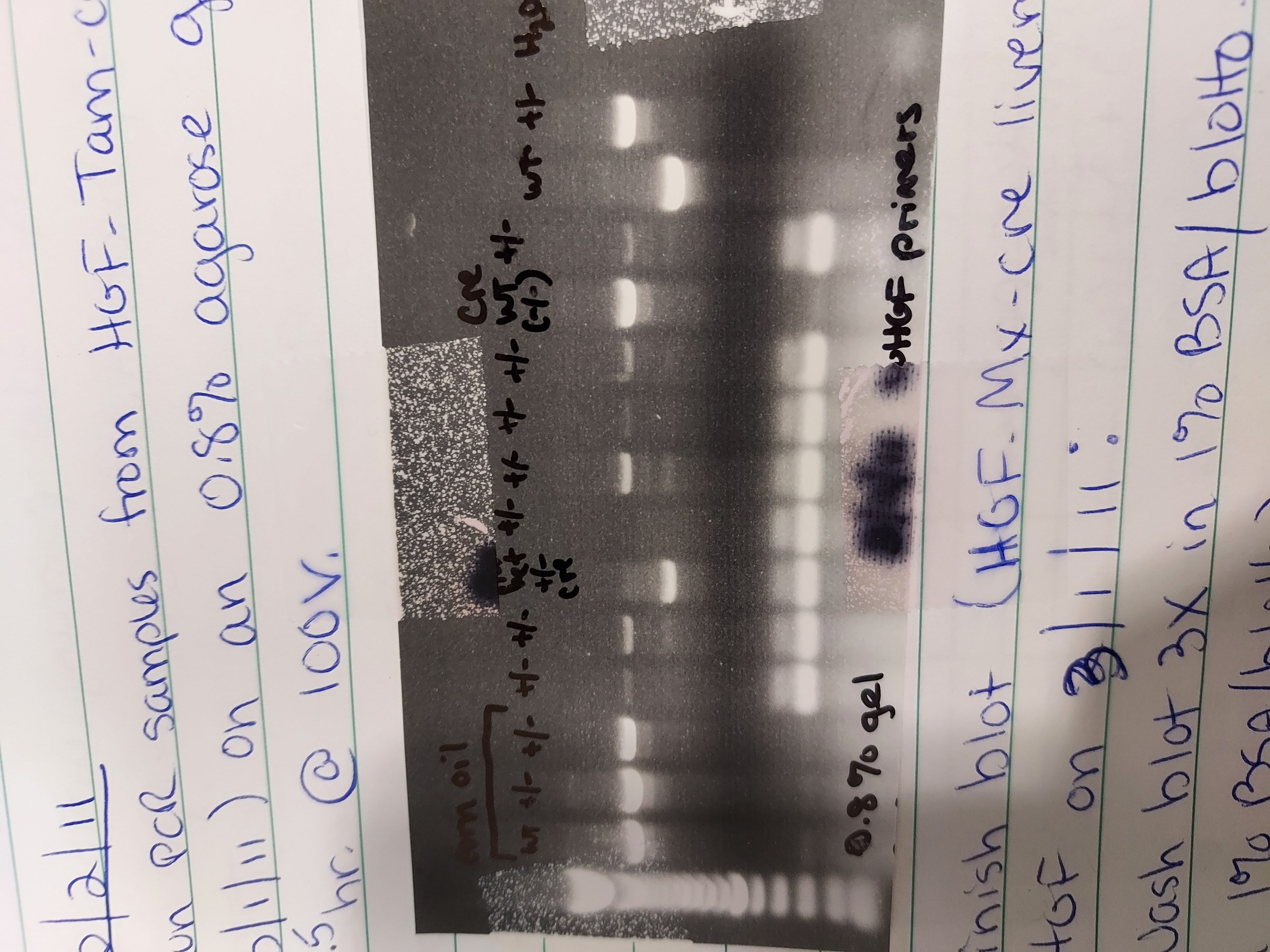

Supplement: S1 File — (ZIP) [file pone.0282358.s001.zip › PLOS ONE images/Fig1Btop-1.jpg]

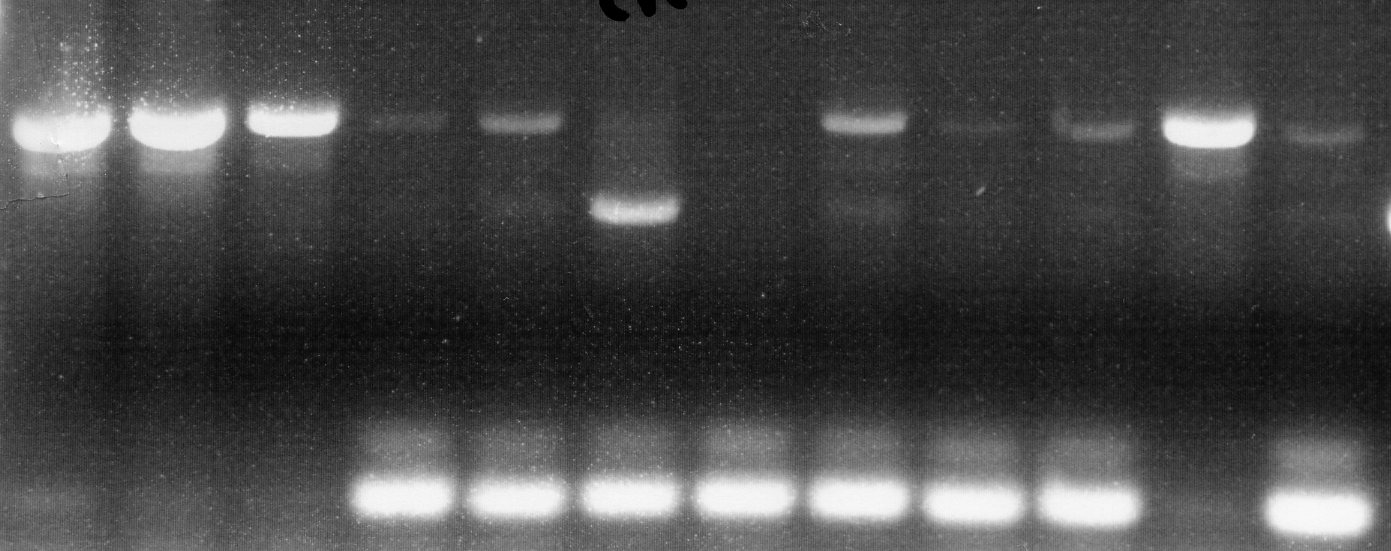

Supplement: S1 File — (ZIP) [file pone.0282358.s001.zip › PLOS ONE images/Fig1Btop-2.jpg]

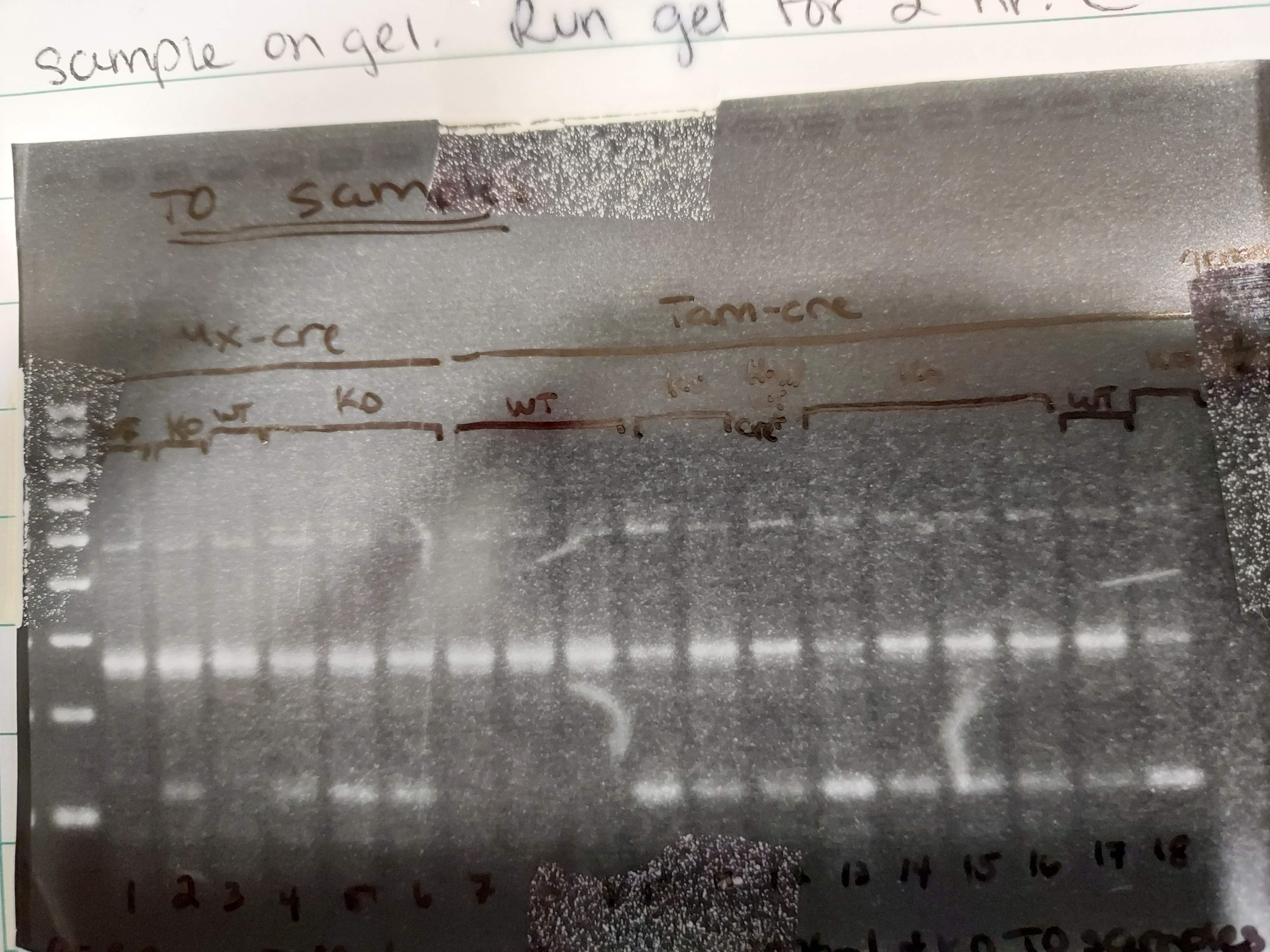

Supplement: S1 File — (ZIP) [file pone.0282358.s001.zip › PLOS ONE images/Fig1C-1.jpg]

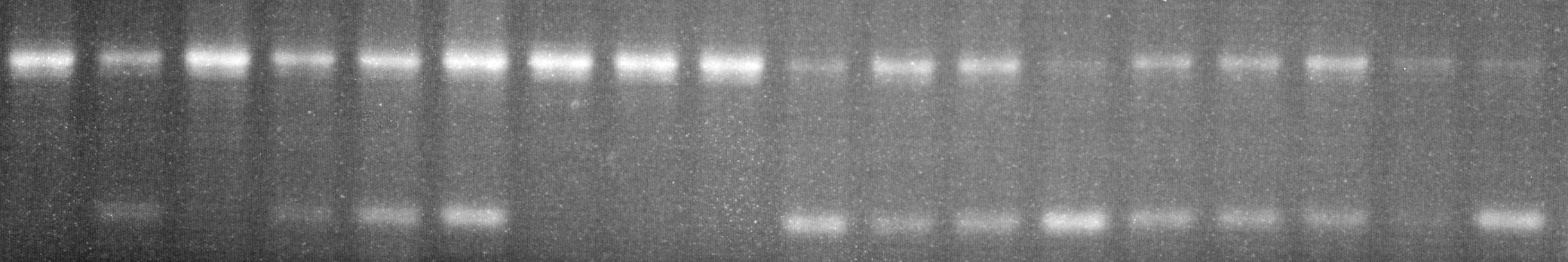

Supplement: S1 File — (ZIP) [file pone.0282358.s001.zip › PLOS ONE images/Fig1C-2.jpg]

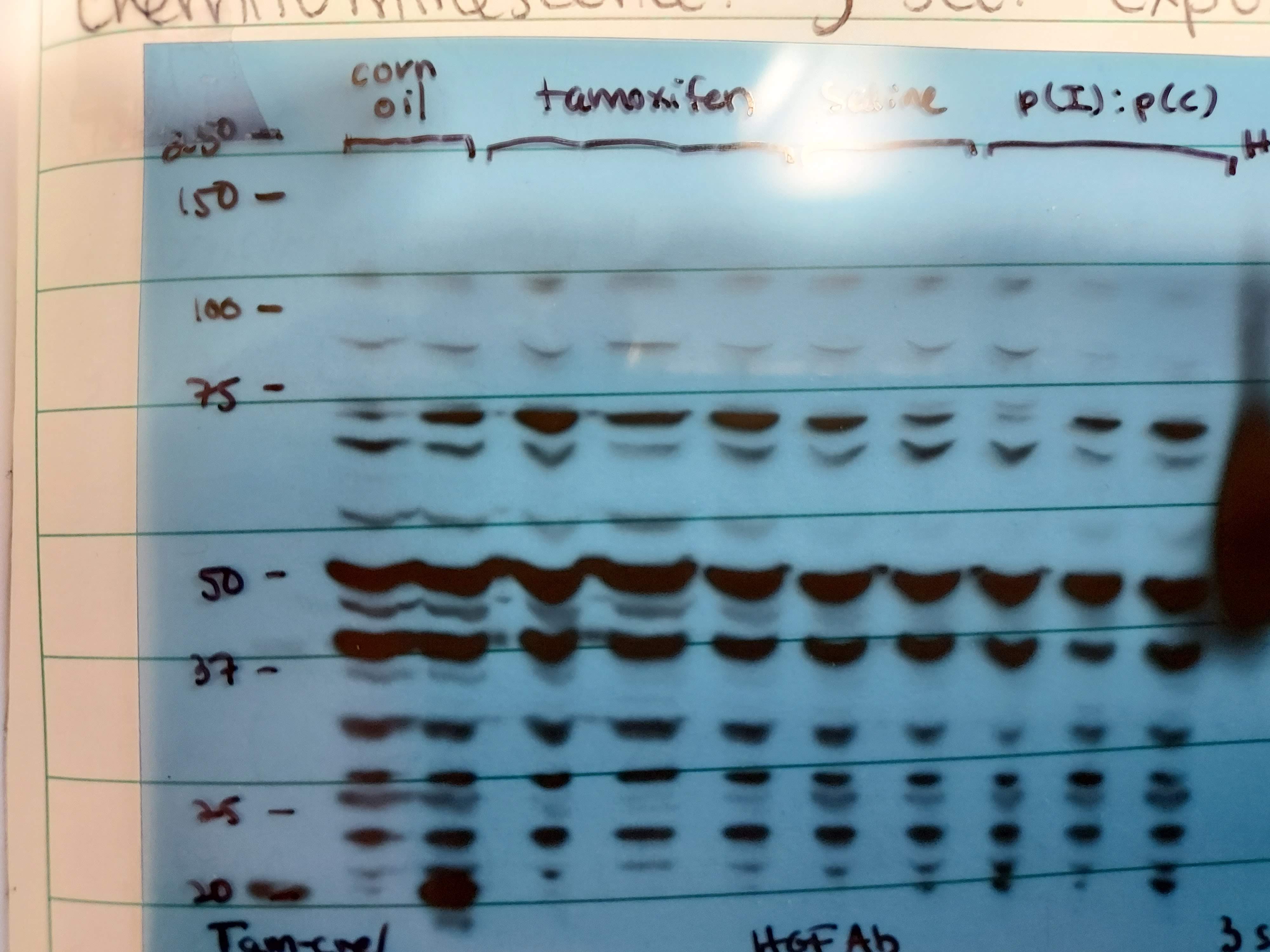

Supplement: S1 File — (ZIP) [file pone.0282358.s001.zip › PLOS ONE images/Fig1D-1.jpg]

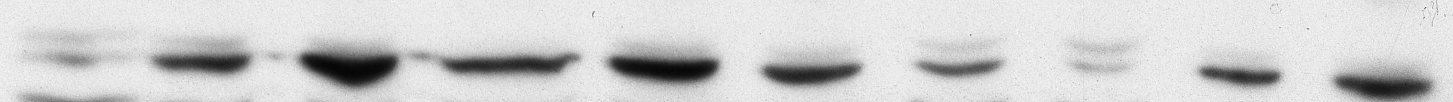

Supplement: S1 File — (ZIP) [file pone.0282358.s001.zip › PLOS ONE images/Fig1D-2.jpg]

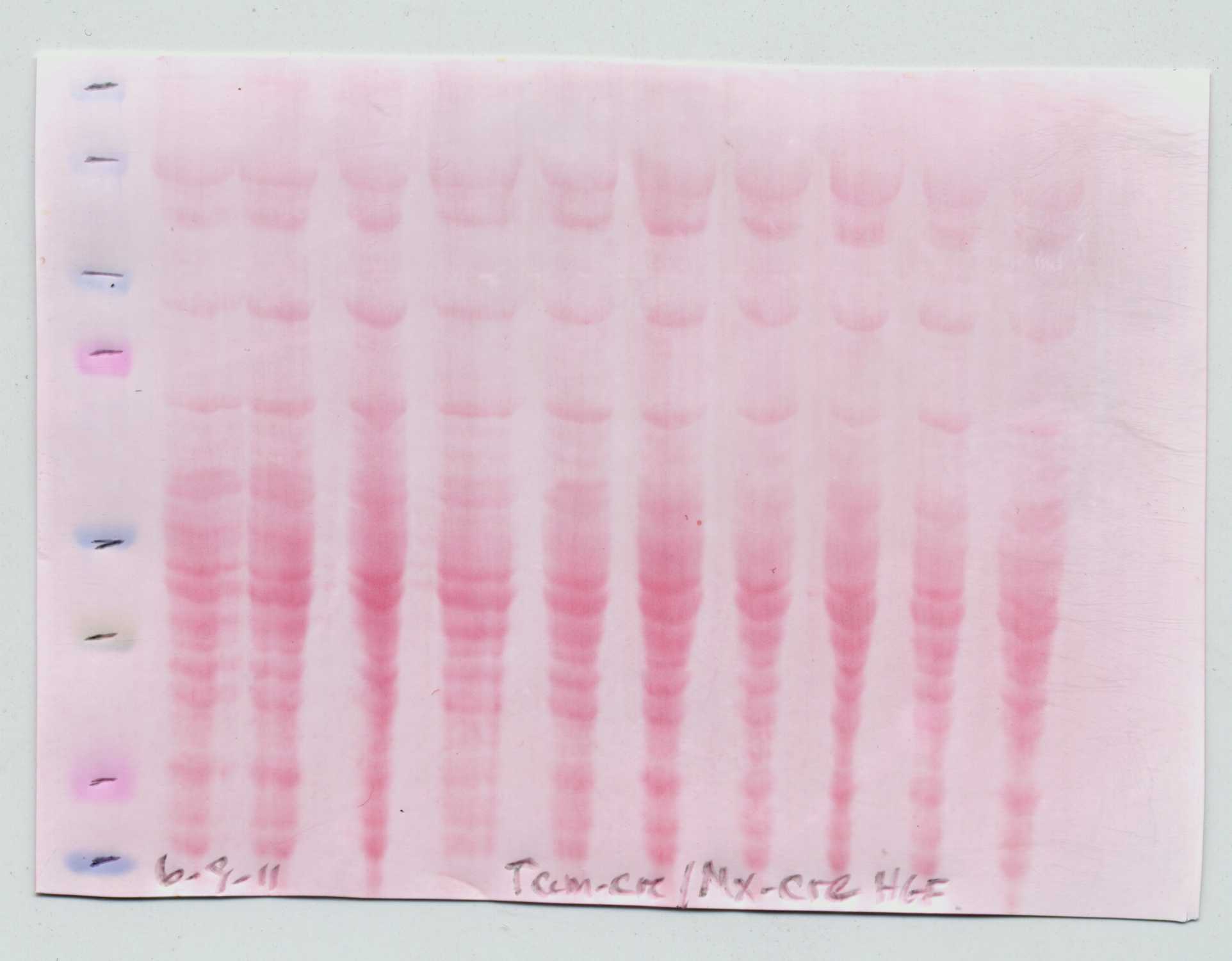

Supplement: S1 File — (ZIP) [file pone.0282358.s001.zip › PLOS ONE images/Fig1D-3.jpg]

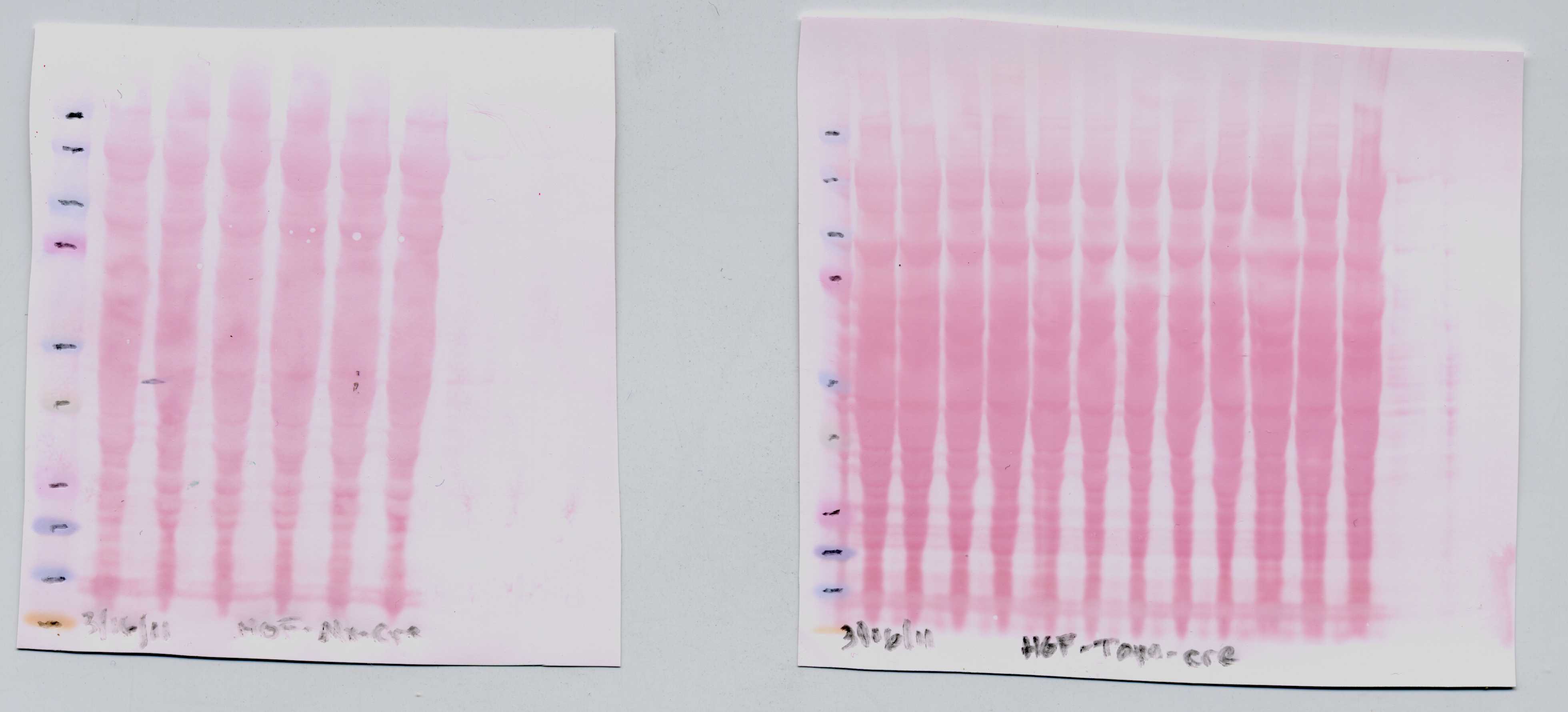

Supplement: S1 File — (ZIP) [file pone.0282358.s001.zip › PLOS ONE images/Fig1DMxandtamcreponceau.jpg]

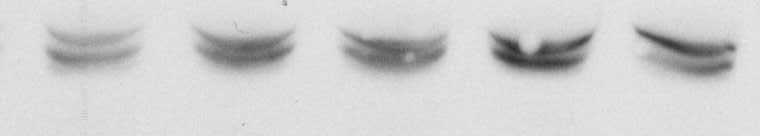

Supplement: S1 File — (ZIP) [file pone.0282358.s001.zip › PLOS ONE images/Fig1Dmxcre.jpg]

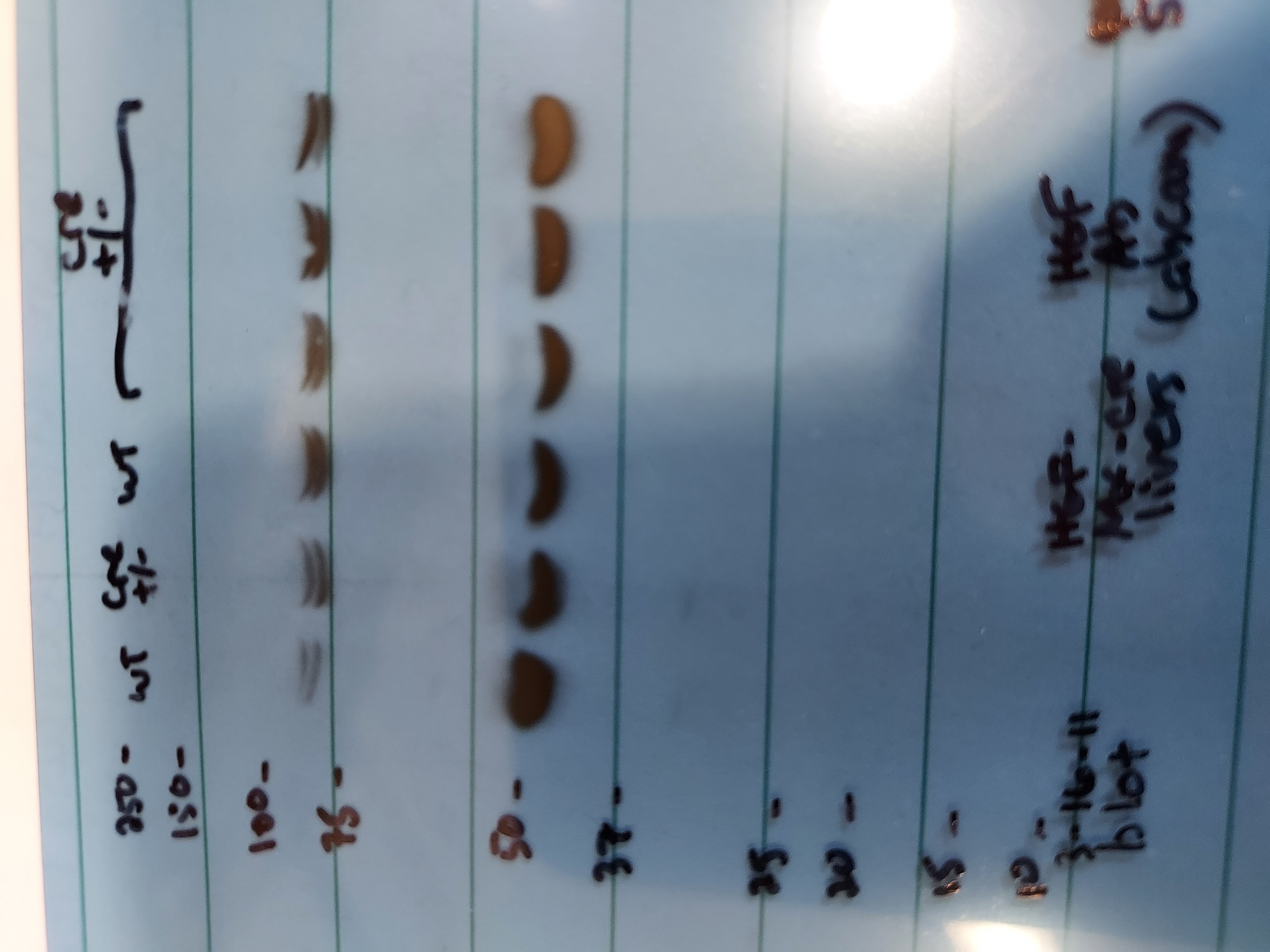

Supplement: S1 File — (ZIP) [file pone.0282358.s001.zip › PLOS ONE images/Fig1Dmxcrewhole.jpg]

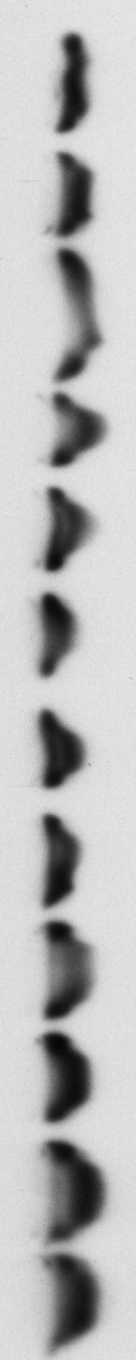

Supplement: S1 File — (ZIP) [file pone.0282358.s001.zip › PLOS ONE images/Fig1Dtamcre.jpg]

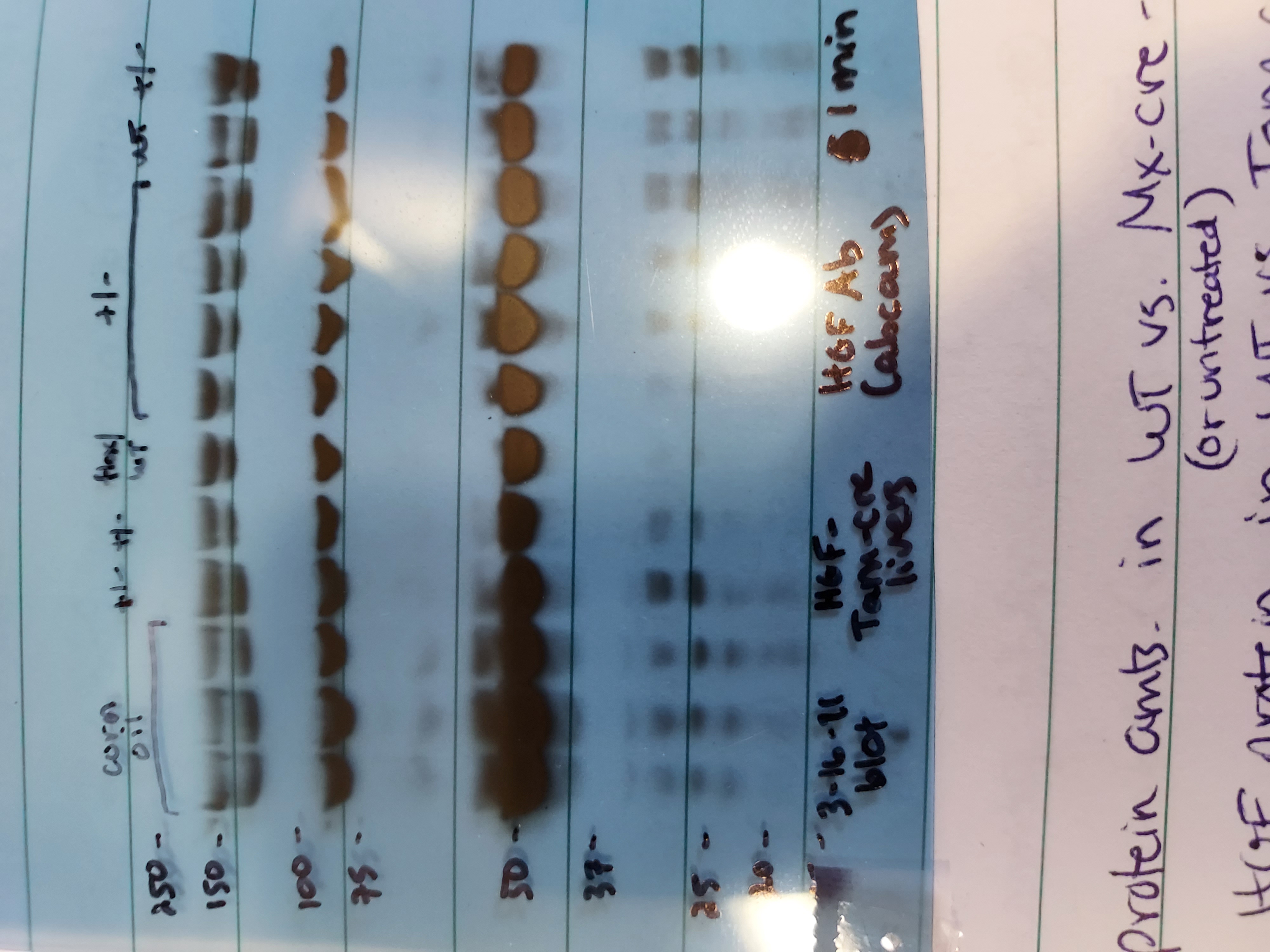

Supplement: S1 File — (ZIP) [file pone.0282358.s001.zip › PLOS ONE images/Fig1Dtamcrewhole.jpg]

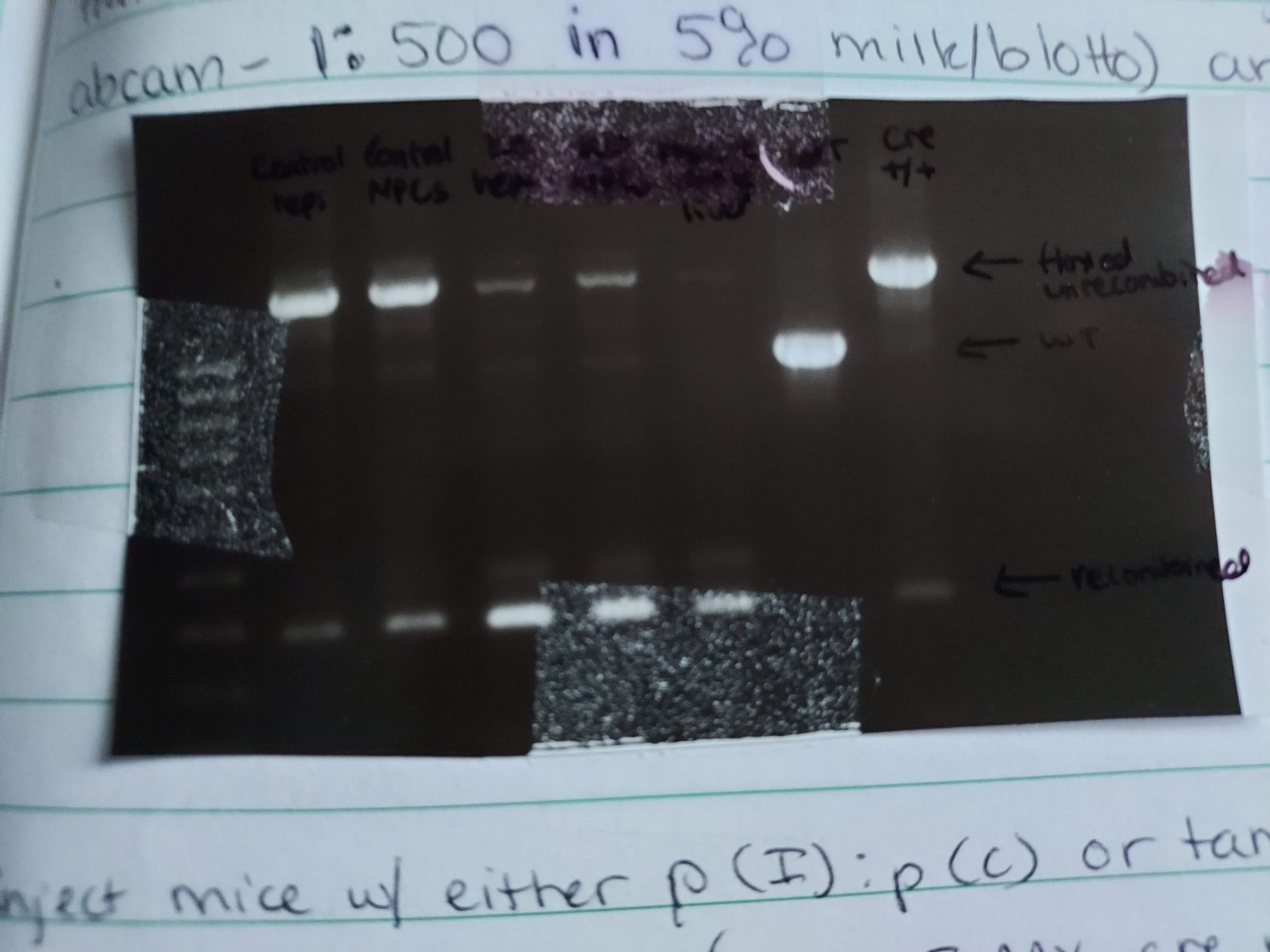

Supplement: S1 File — (ZIP) [file pone.0282358.s001.zip › PLOS ONE images/Fig2A-1.jpg]

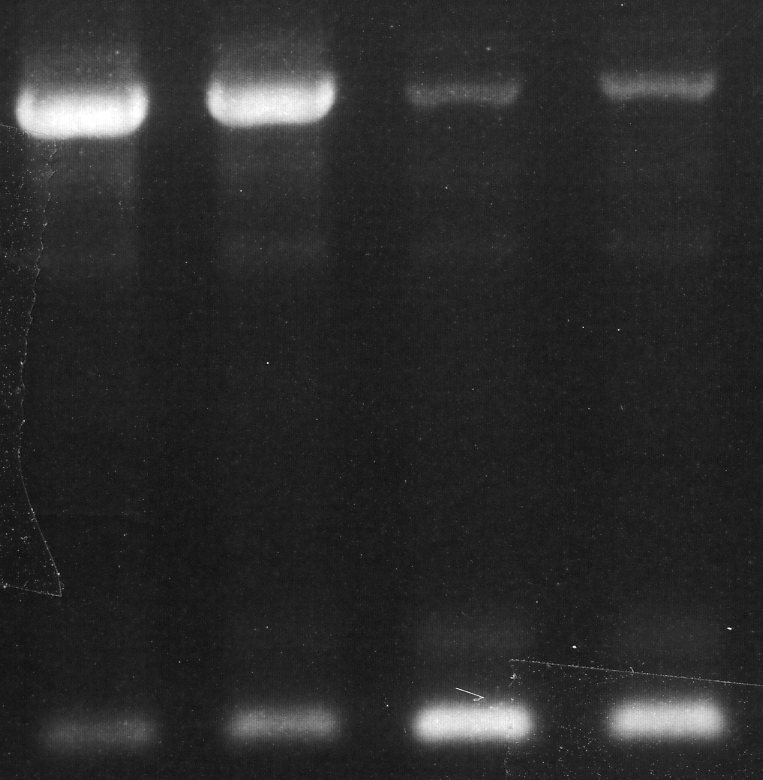

Supplement: S1 File — (ZIP) [file pone.0282358.s001.zip › PLOS ONE images/Fig2A-2.jpg]

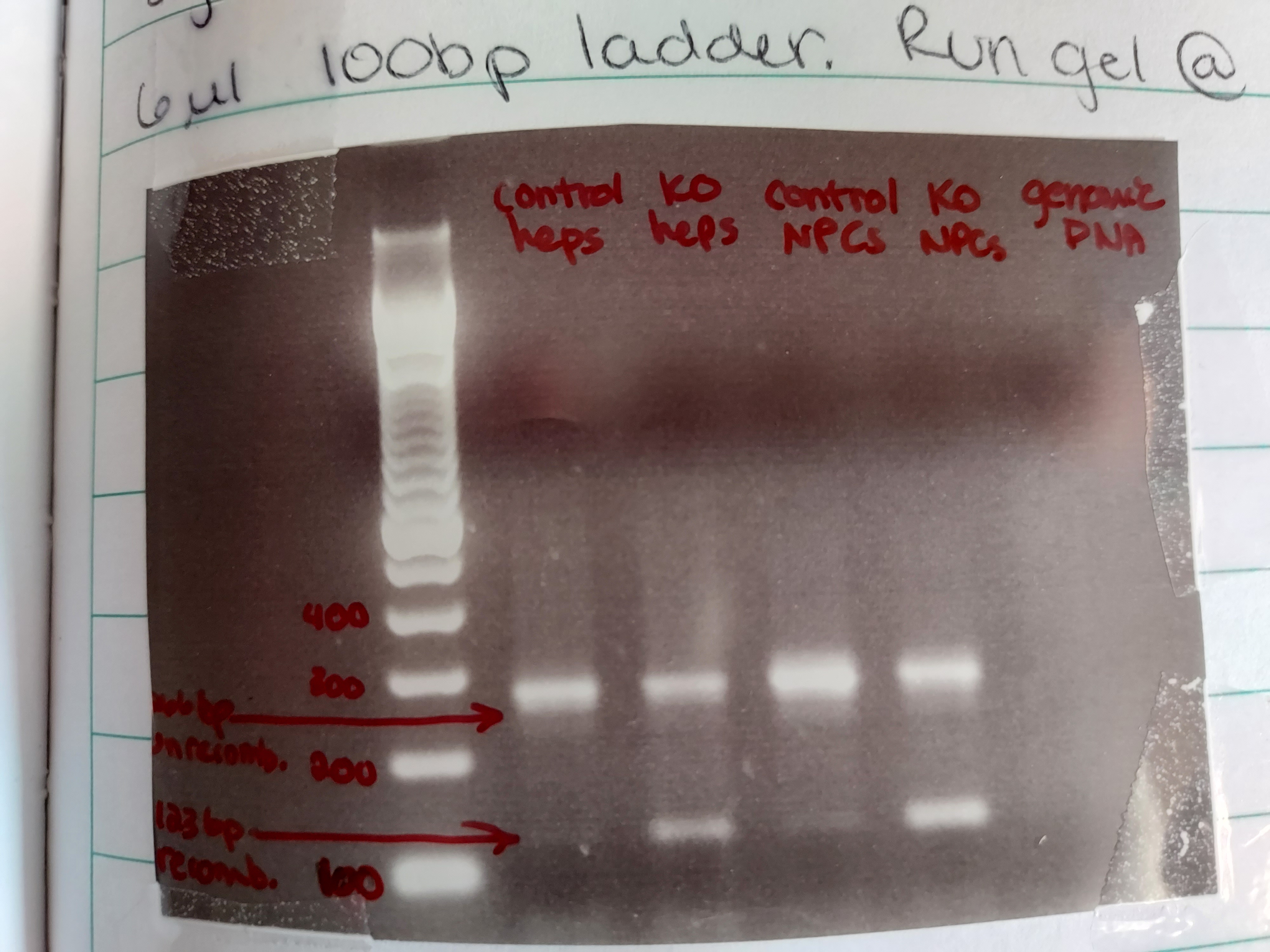

Supplement: S1 File — (ZIP) [file pone.0282358.s001.zip › PLOS ONE images/Fig2B-1.jpg]

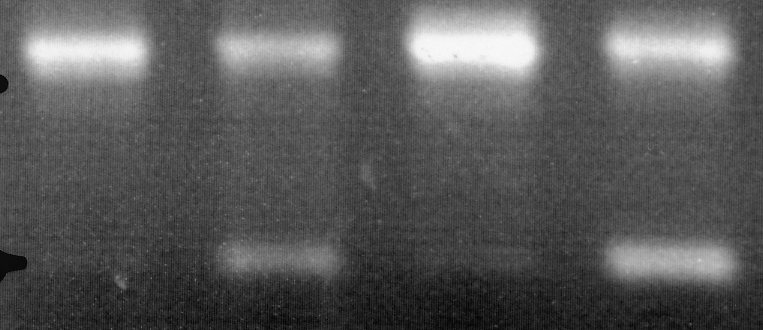

Supplement: S1 File — (ZIP) [file pone.0282358.s001.zip › PLOS ONE images/Fig2B-2.jpg]

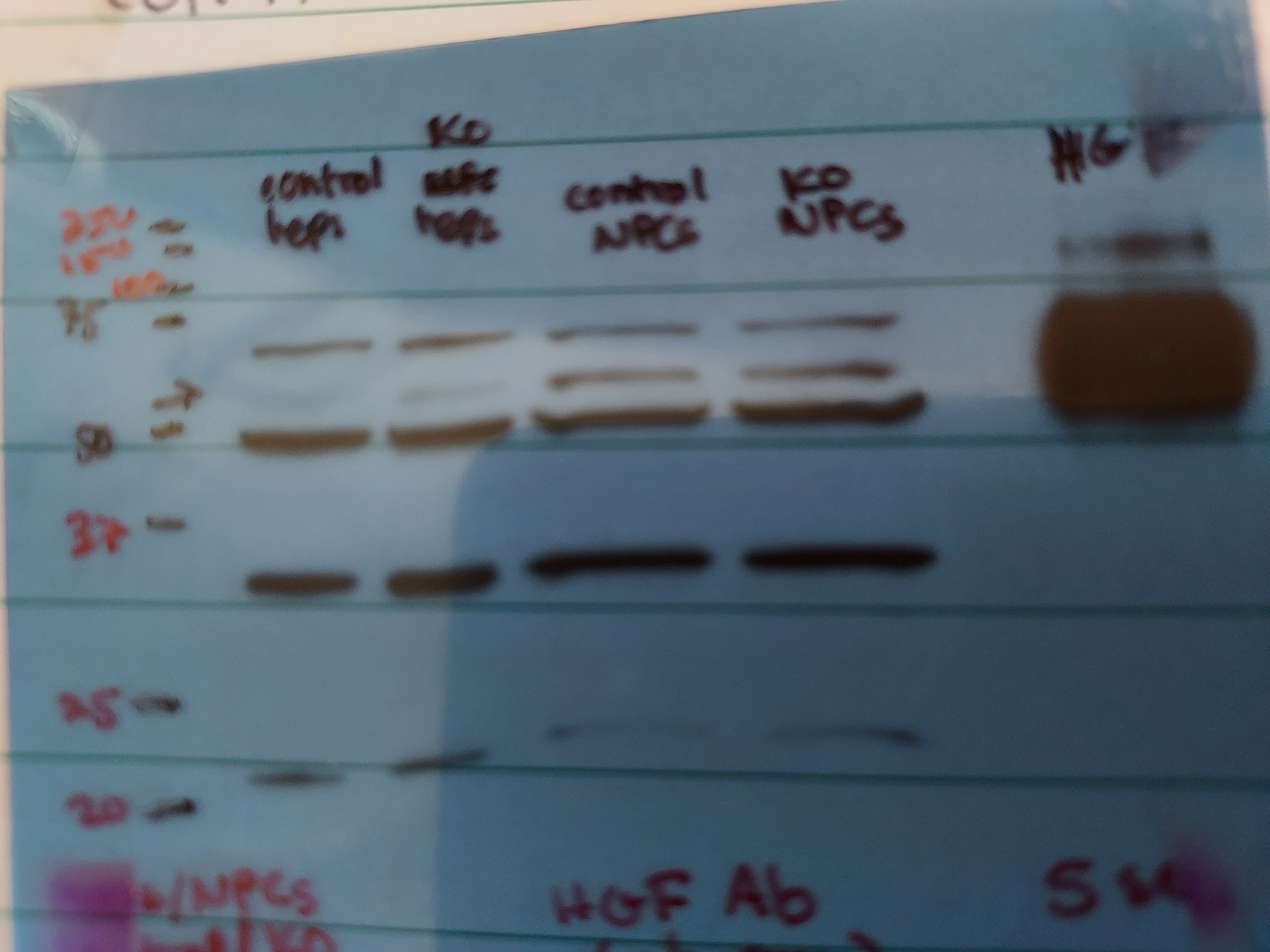

Supplement: S1 File — (ZIP) [file pone.0282358.s001.zip › PLOS ONE images/Fig2D-1.jpg]

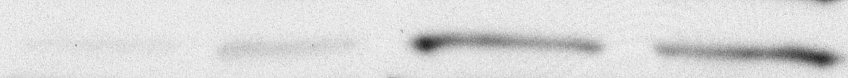

Supplement: S1 File — (ZIP) [file pone.0282358.s001.zip › PLOS ONE images/Fig2D-2.jpg]

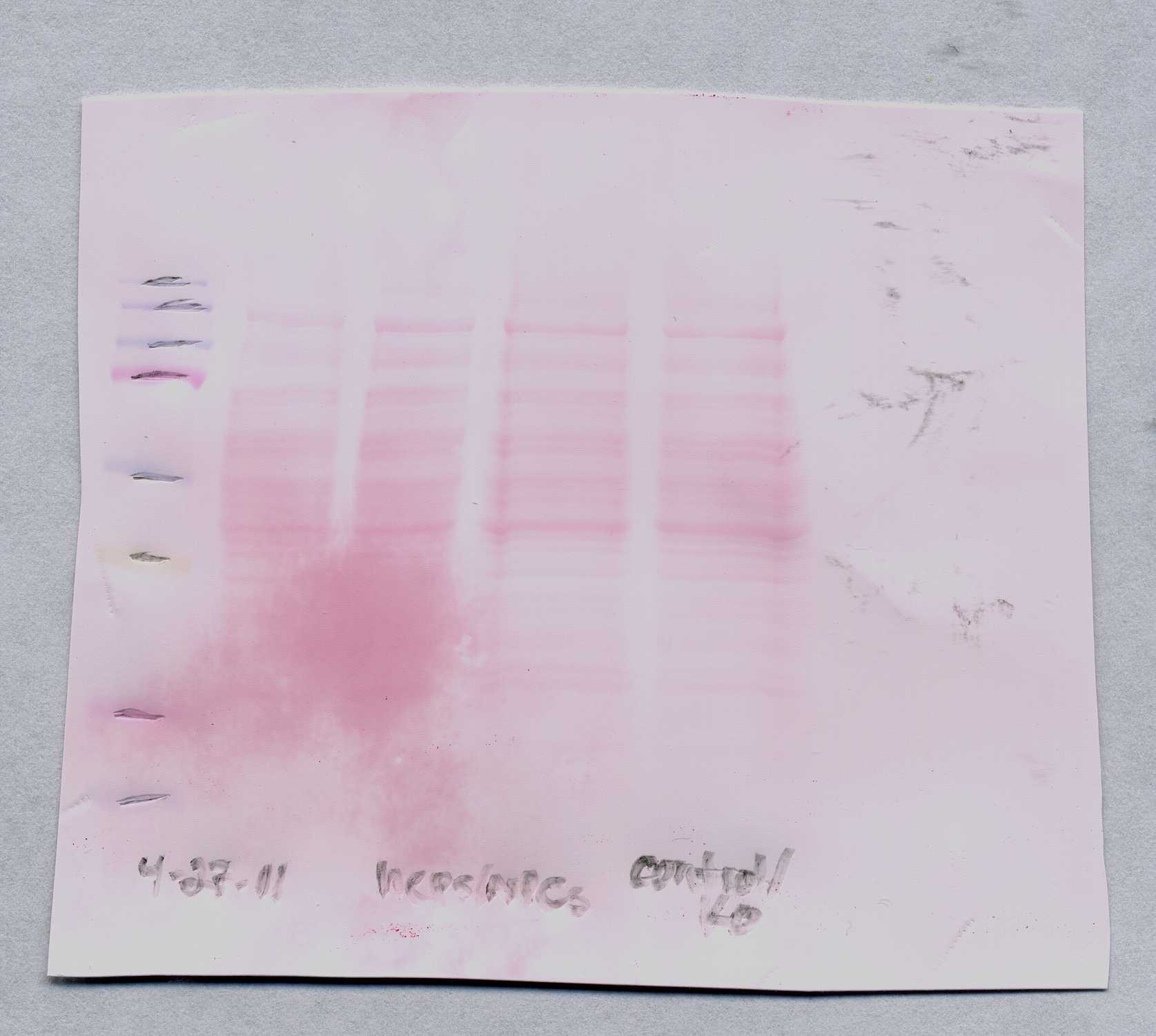

Supplement: S1 File — (ZIP) [file pone.0282358.s001.zip › PLOS ONE images/Fig2Dponceau.jpg]

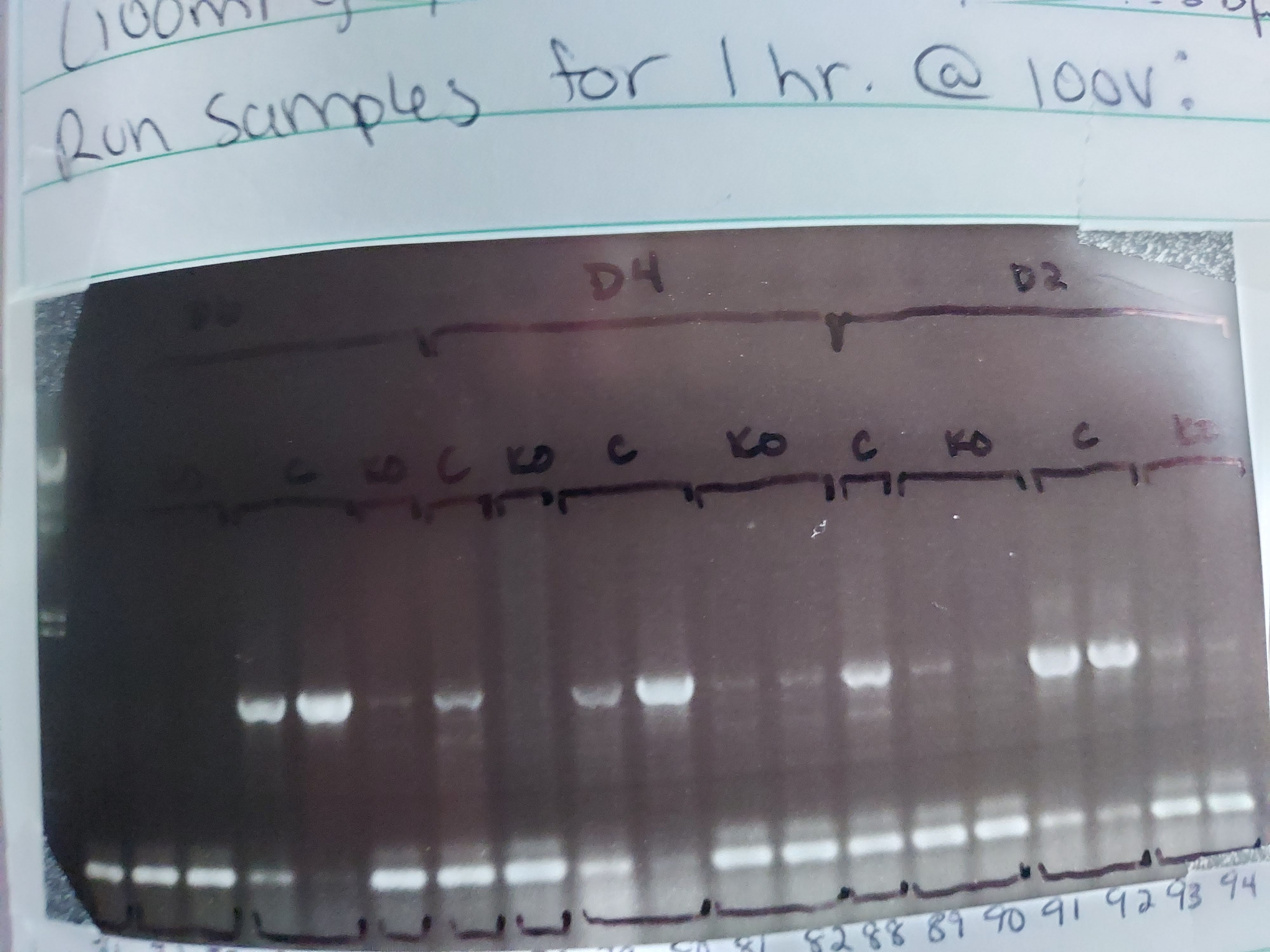

Supplement: S1 File — (ZIP) [file pone.0282358.s001.zip › PLOS ONE images/Fig3A-1.jpg]

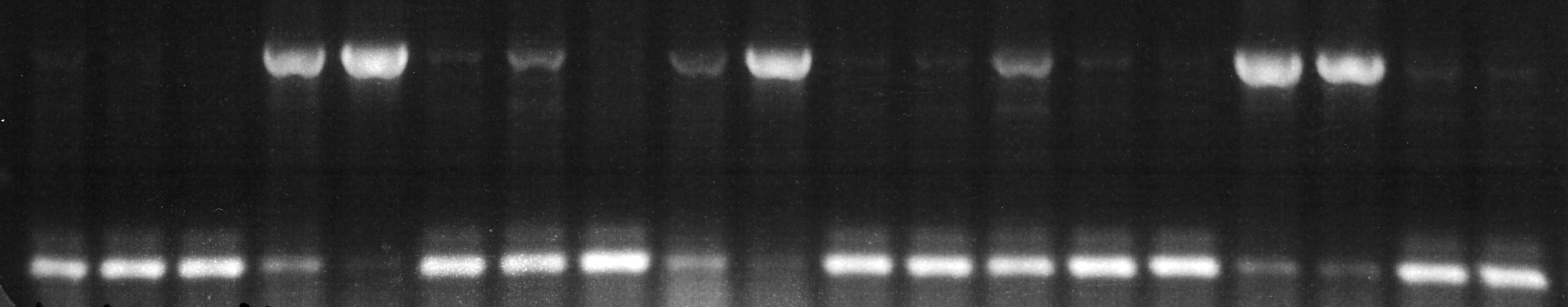

Supplement: S1 File — (ZIP) [file pone.0282358.s001.zip › PLOS ONE images/Fig3A-2.jpg]

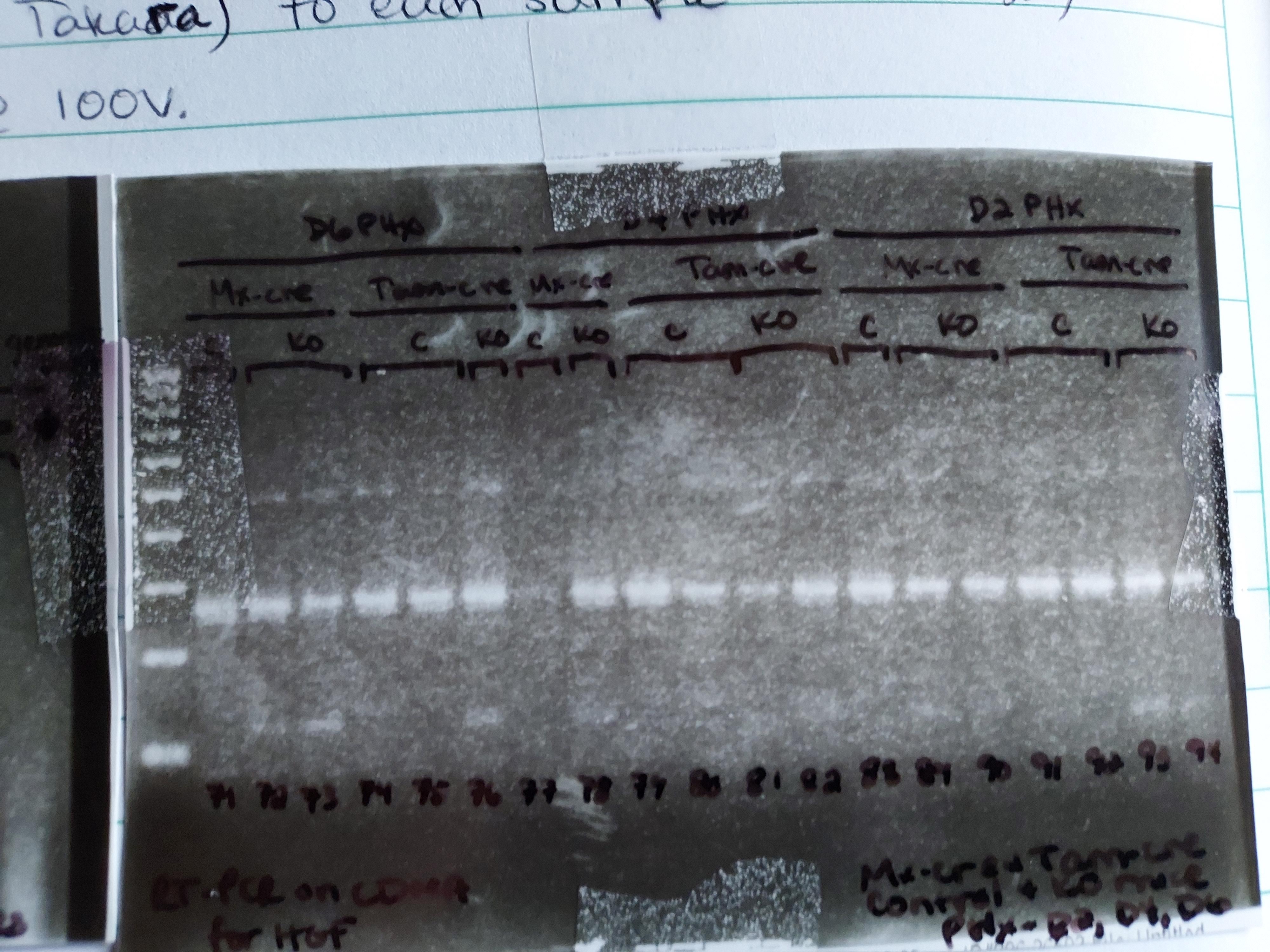

Supplement: S1 File — (ZIP) [file pone.0282358.s001.zip › PLOS ONE images/Fig3B-1.jpg]

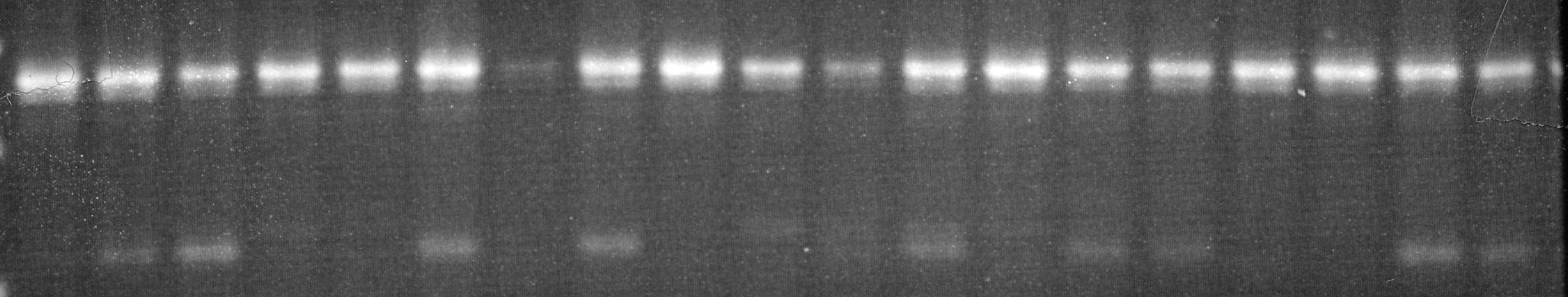

Supplement: S1 File — (ZIP) [file pone.0282358.s001.zip › PLOS ONE images/Fig3B-2.jpg]

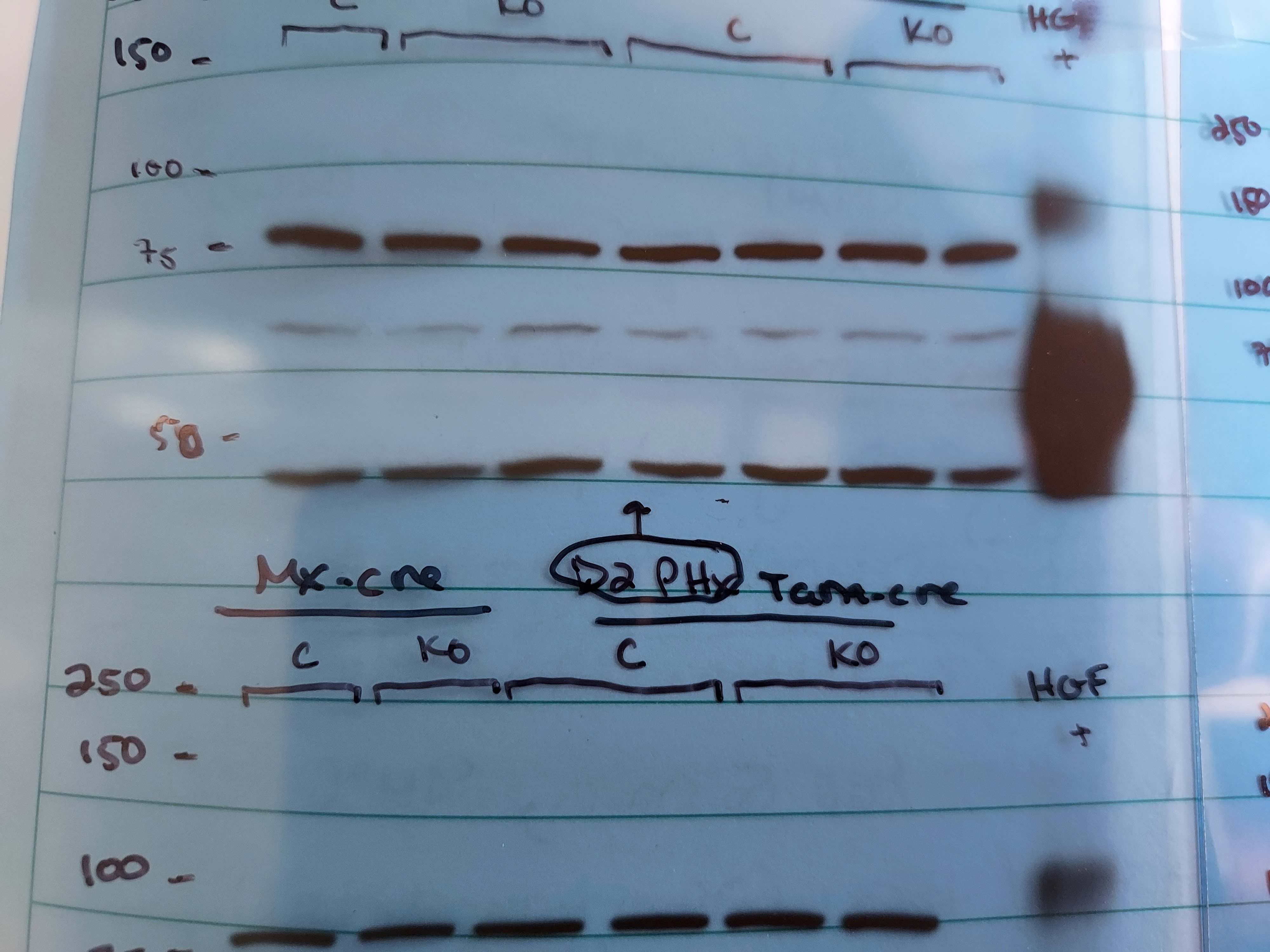

Supplement: S1 File — (ZIP) [file pone.0282358.s001.zip › PLOS ONE images/Fig3D-1.jpg]

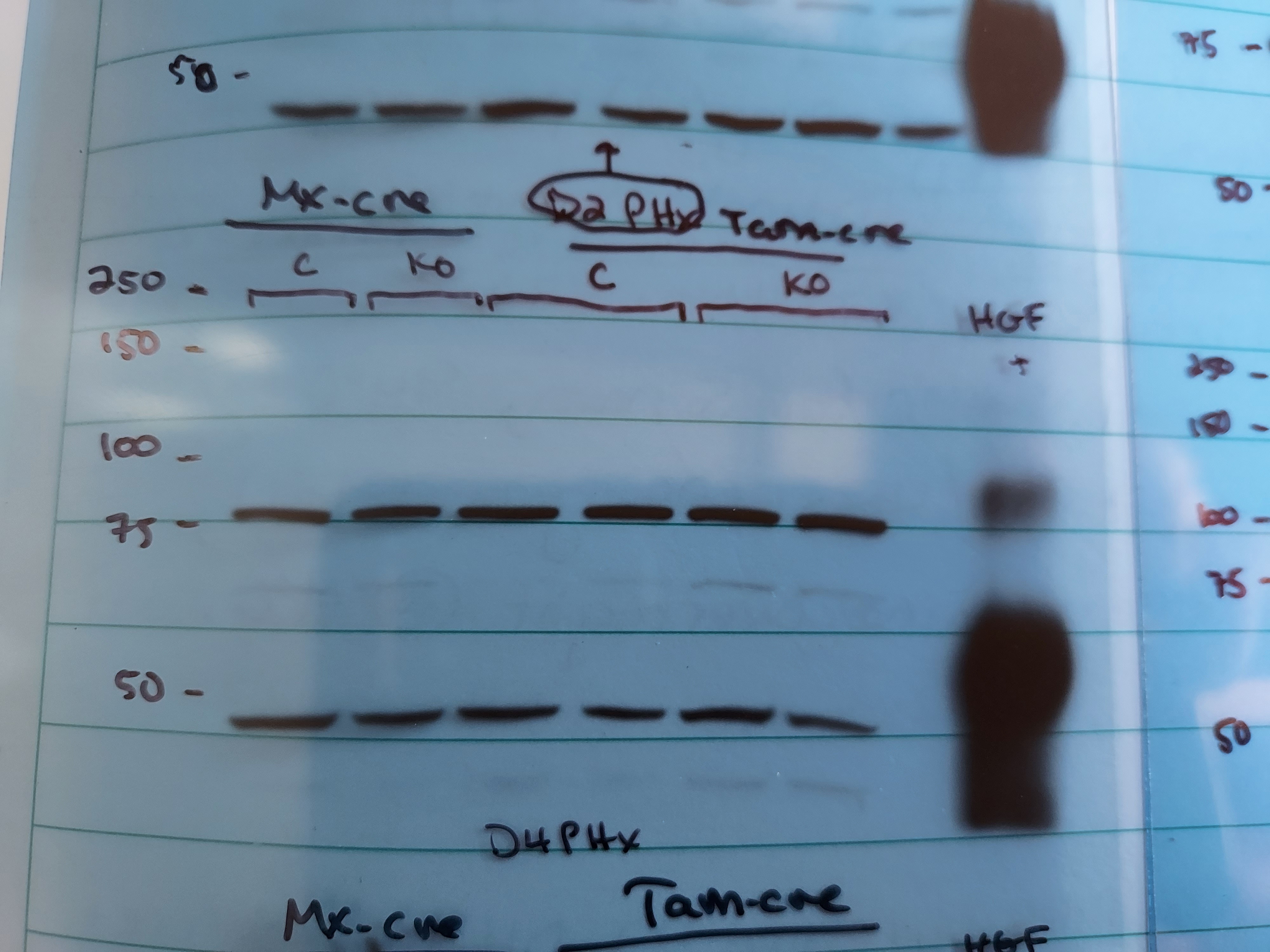

Supplement: S1 File — (ZIP) [file pone.0282358.s001.zip › PLOS ONE images/Fig3D-2.jpg]

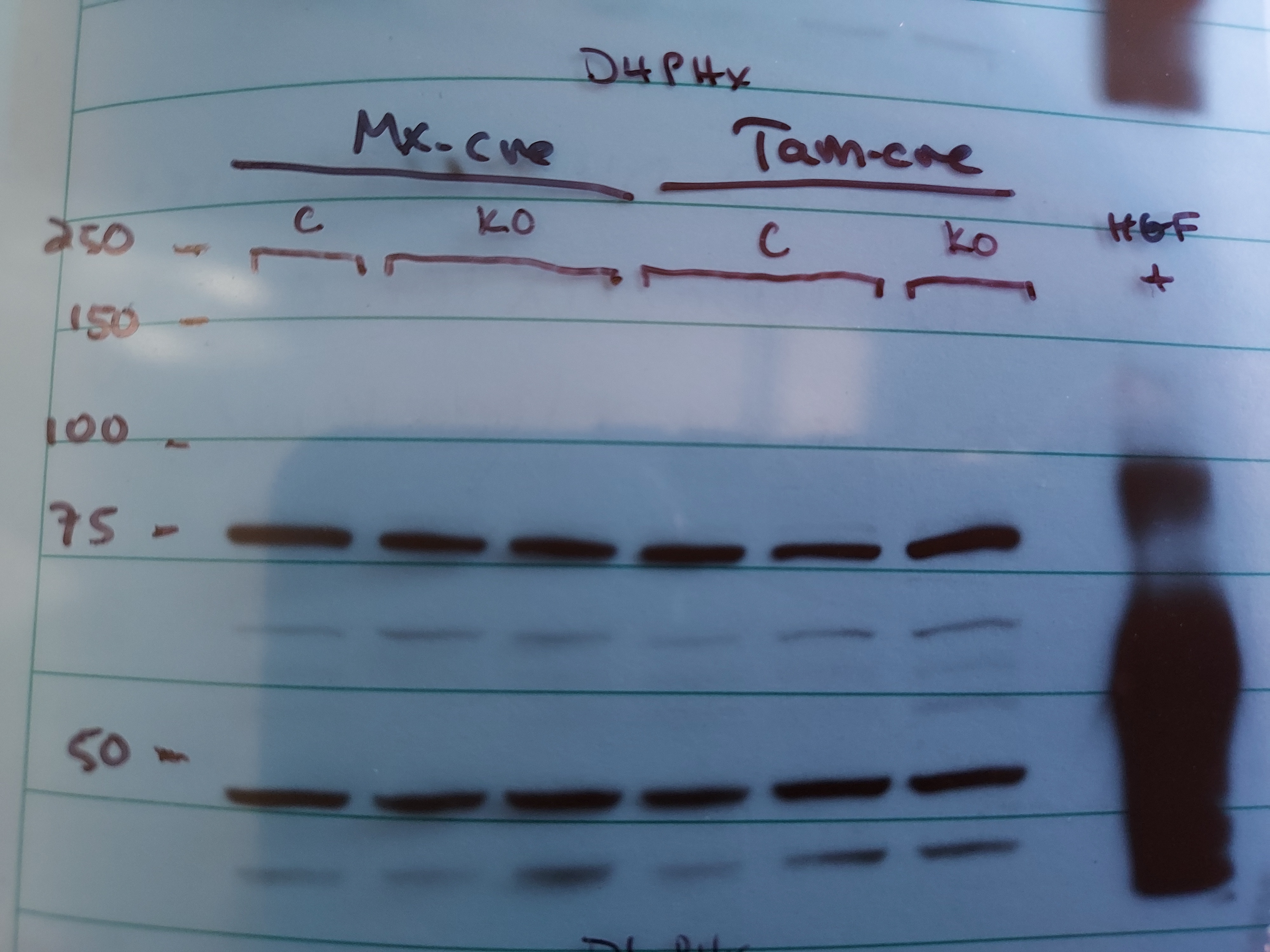

Supplement: S1 File — (ZIP) [file pone.0282358.s001.zip › PLOS ONE images/Fig3D-3.jpg]

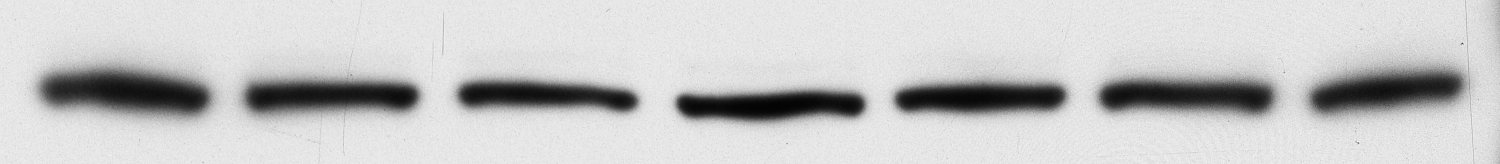

Supplement: S1 File — (ZIP) [file pone.0282358.s001.zip › PLOS ONE images/Fig3D-4.jpg]

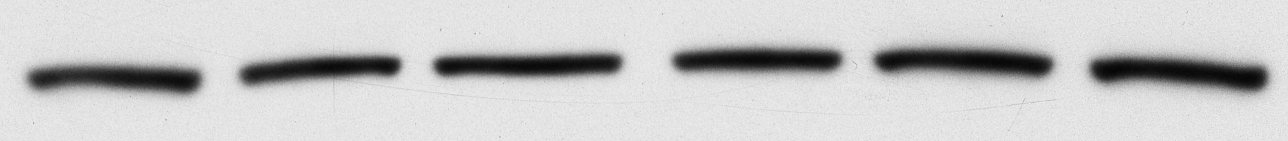

Supplement: S1 File — (ZIP) [file pone.0282358.s001.zip › PLOS ONE images/Fig3D-5.jpg]

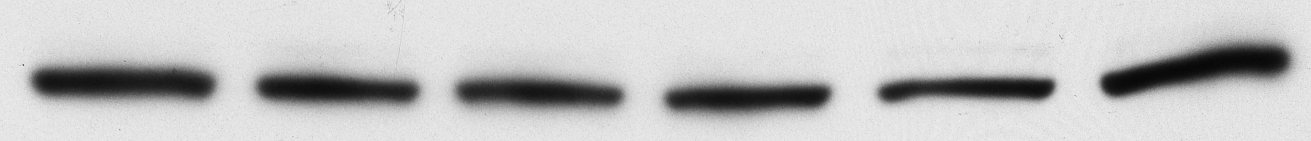

Supplement: S1 File — (ZIP) [file pone.0282358.s001.zip › PLOS ONE images/Fig3D-6.jpg]

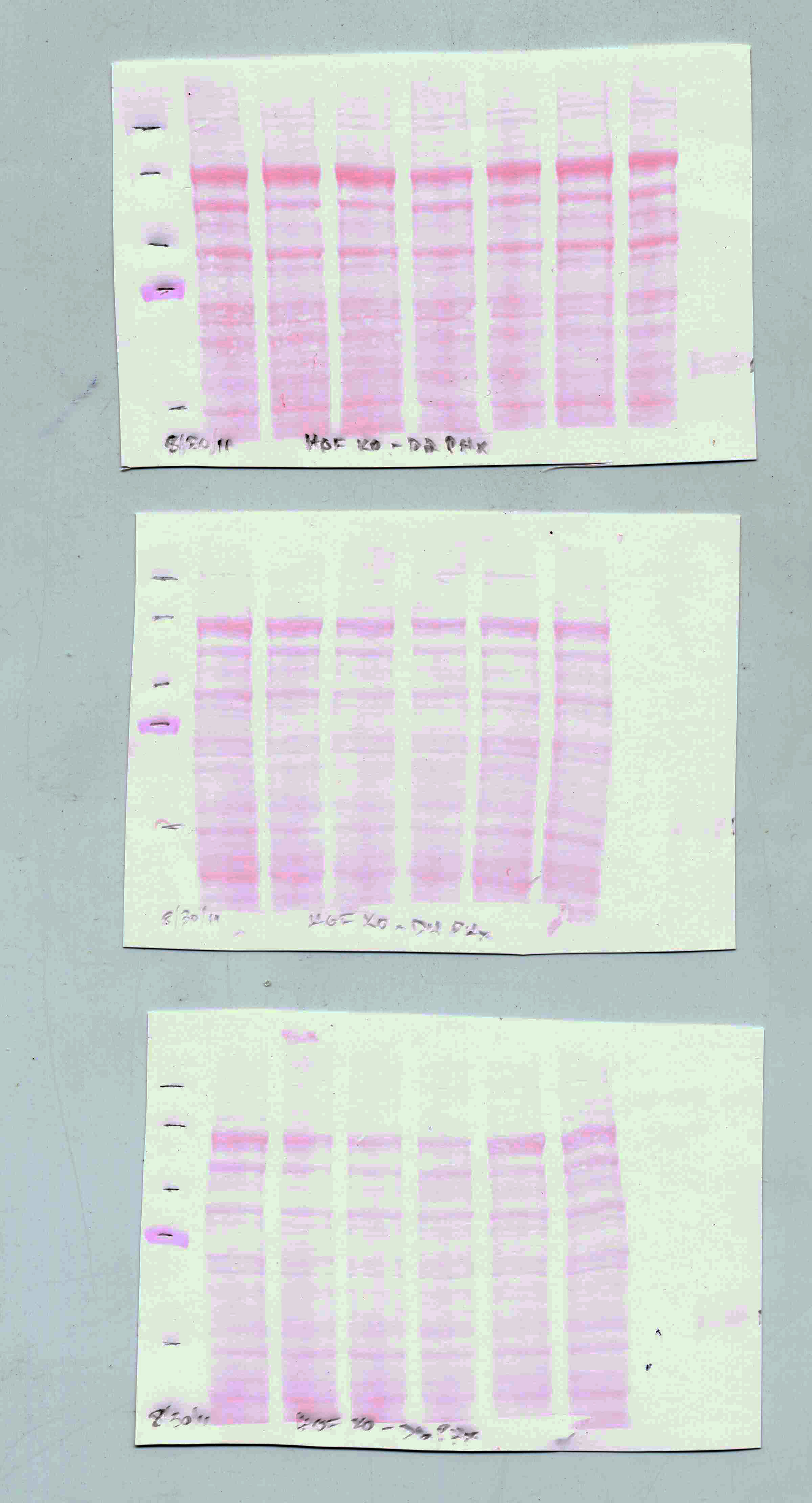

Supplement: S1 File — (ZIP) [file pone.0282358.s001.zip › PLOS ONE images/Fig3Dponceau.jpg]

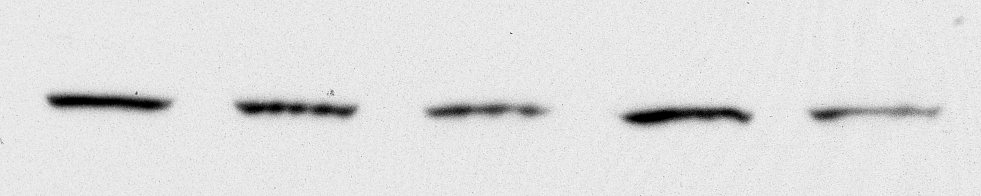

Supplement: S1 File — (ZIP) [file pone.0282358.s001.zip › PLOS ONE images/Fig4B-1.jpg]

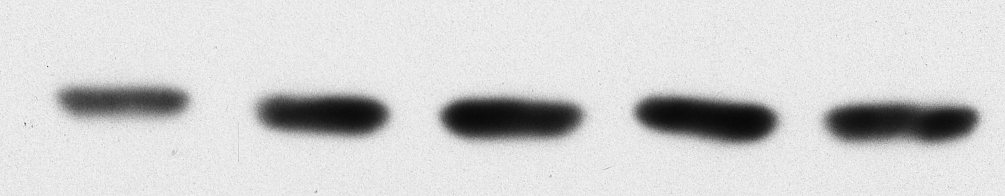

Supplement: S1 File — (ZIP) [file pone.0282358.s001.zip › PLOS ONE images/Fig4B-2.jpg]

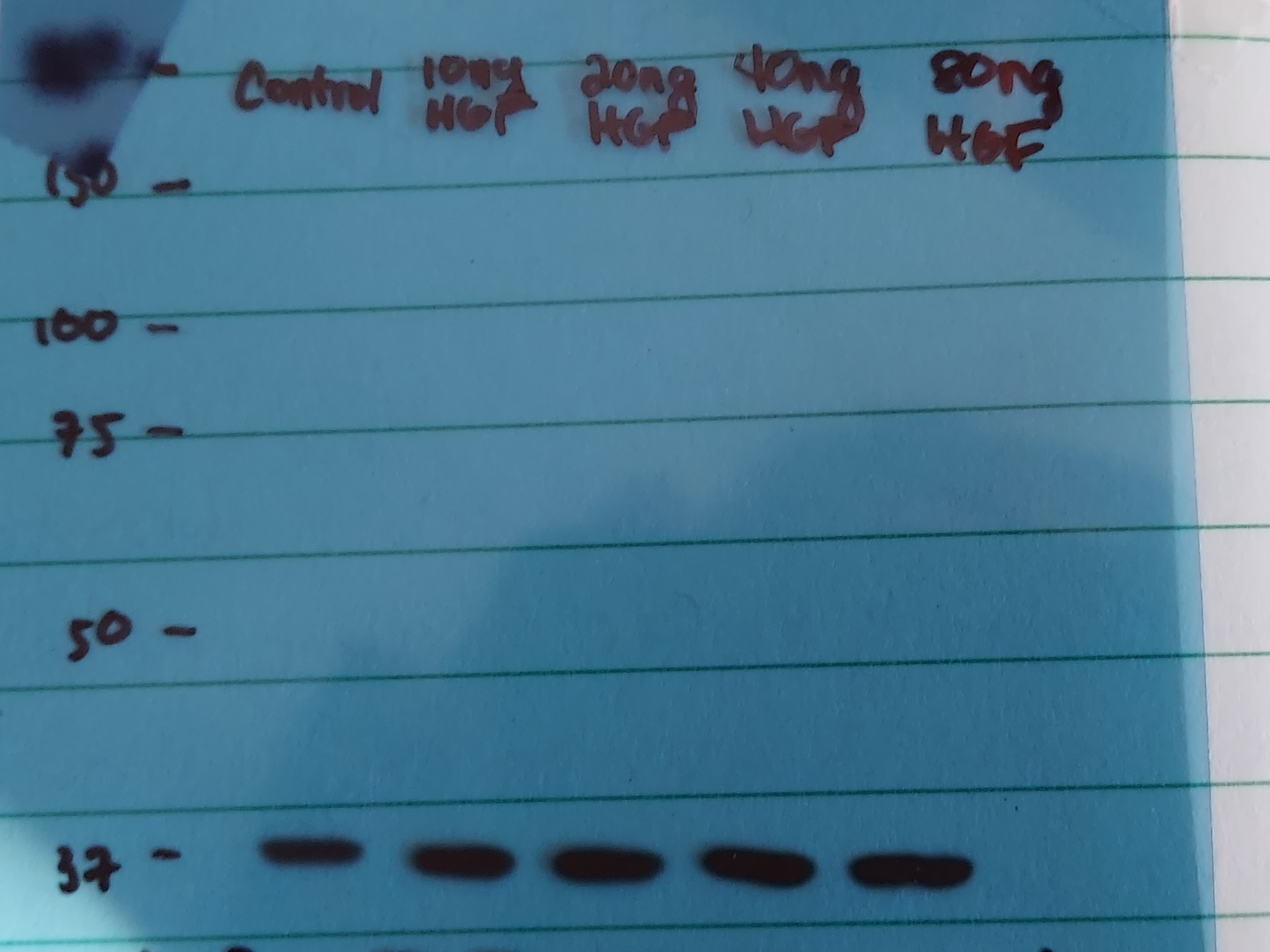

Supplement: S1 File — (ZIP) [file pone.0282358.s001.zip › PLOS ONE images/Fig4Bactinwhole.jpg]

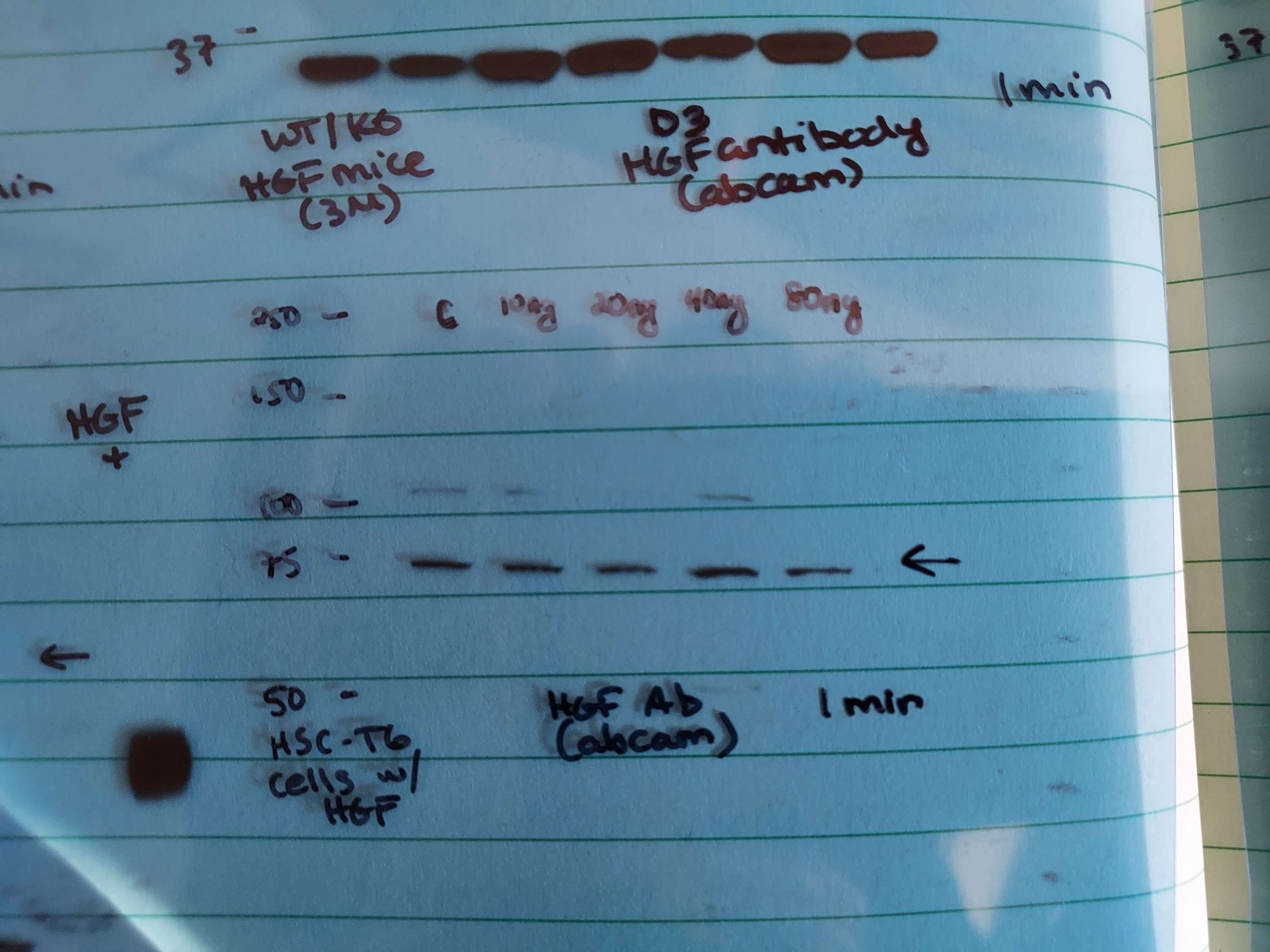

Supplement: S1 File — (ZIP) [file pone.0282358.s001.zip › PLOS ONE images/Fig4BHGFwhole.jpg]

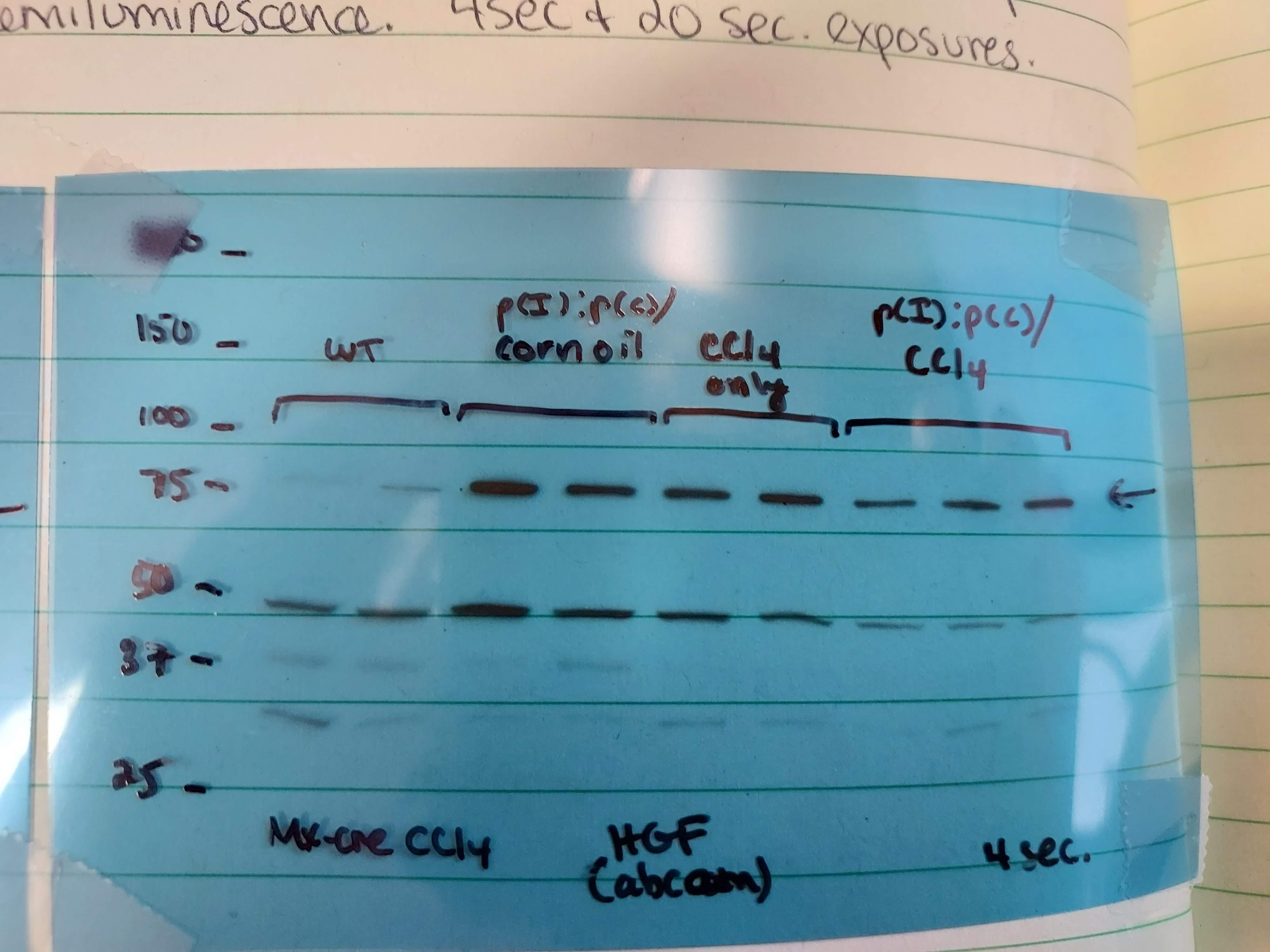

Supplement: S1 File — (ZIP) [file pone.0282358.s001.zip › PLOS ONE images/Fig5B-1.jpg]

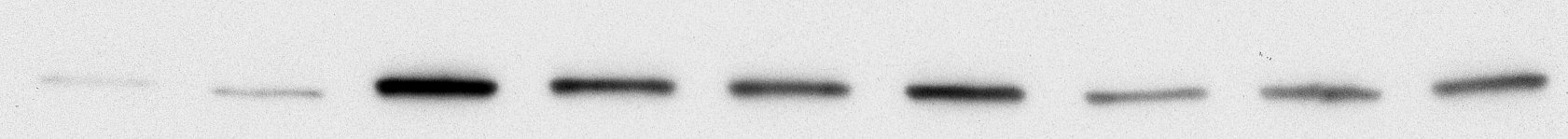

Supplement: S1 File — (ZIP) [file pone.0282358.s001.zip › PLOS ONE images/Fig5B-2.jpg]

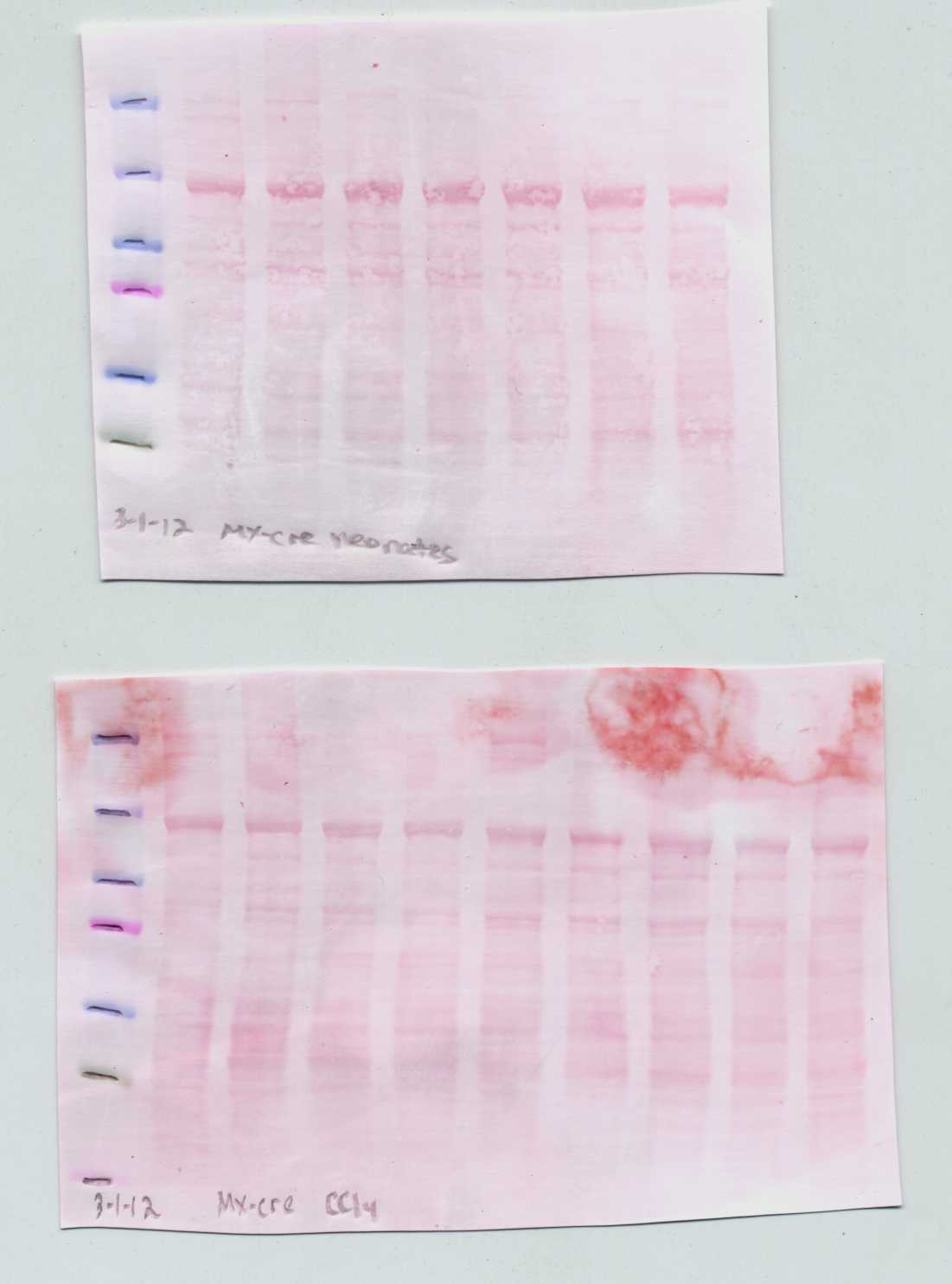

Supplement: S1 File — (ZIP) [file pone.0282358.s001.zip › PLOS ONE images/Fig5Bponceau.jpg]

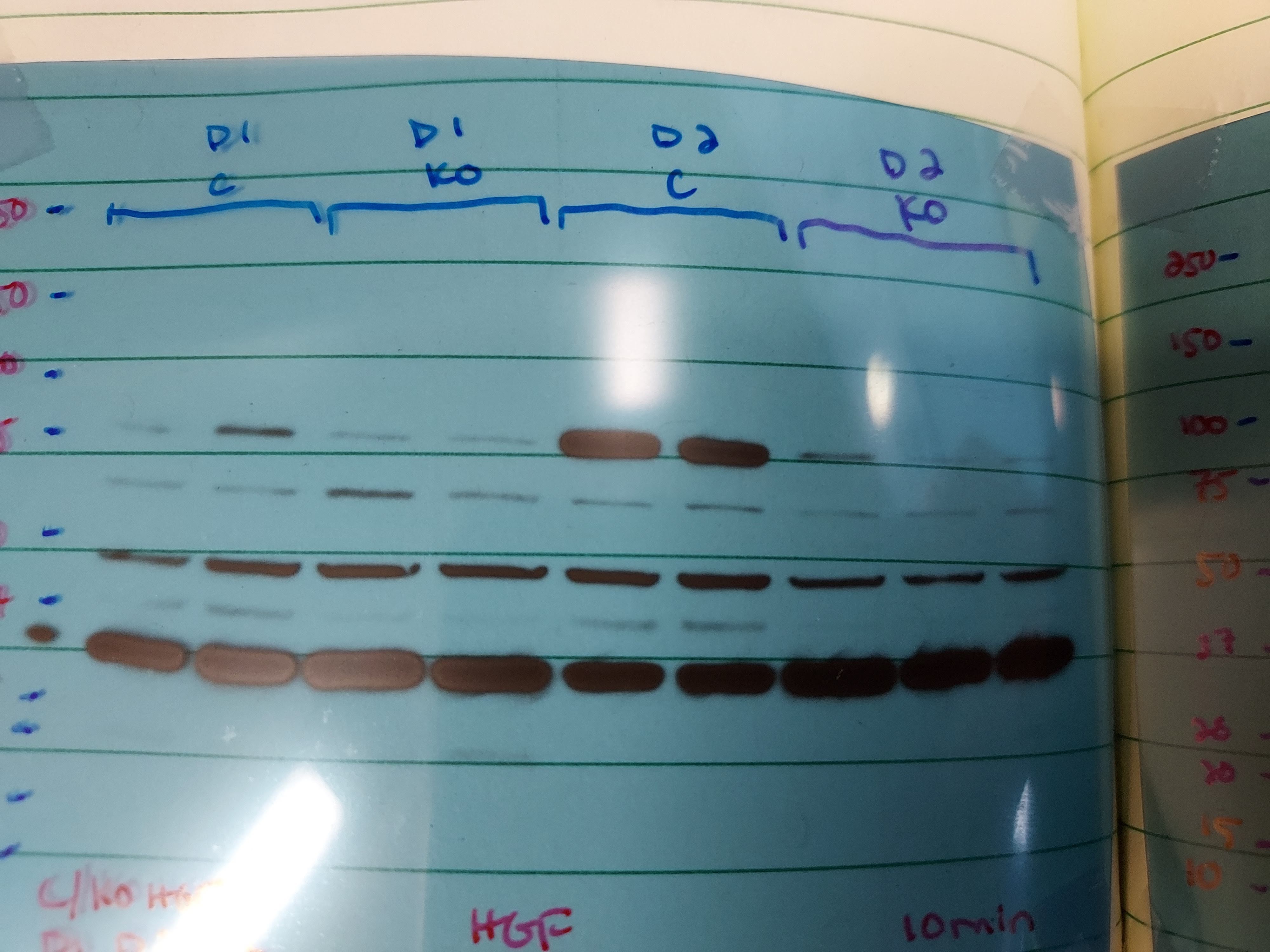

Supplement: S1 File — (ZIP) [file pone.0282358.s001.zip › PLOS ONE images/Fig6E-1.jpg]

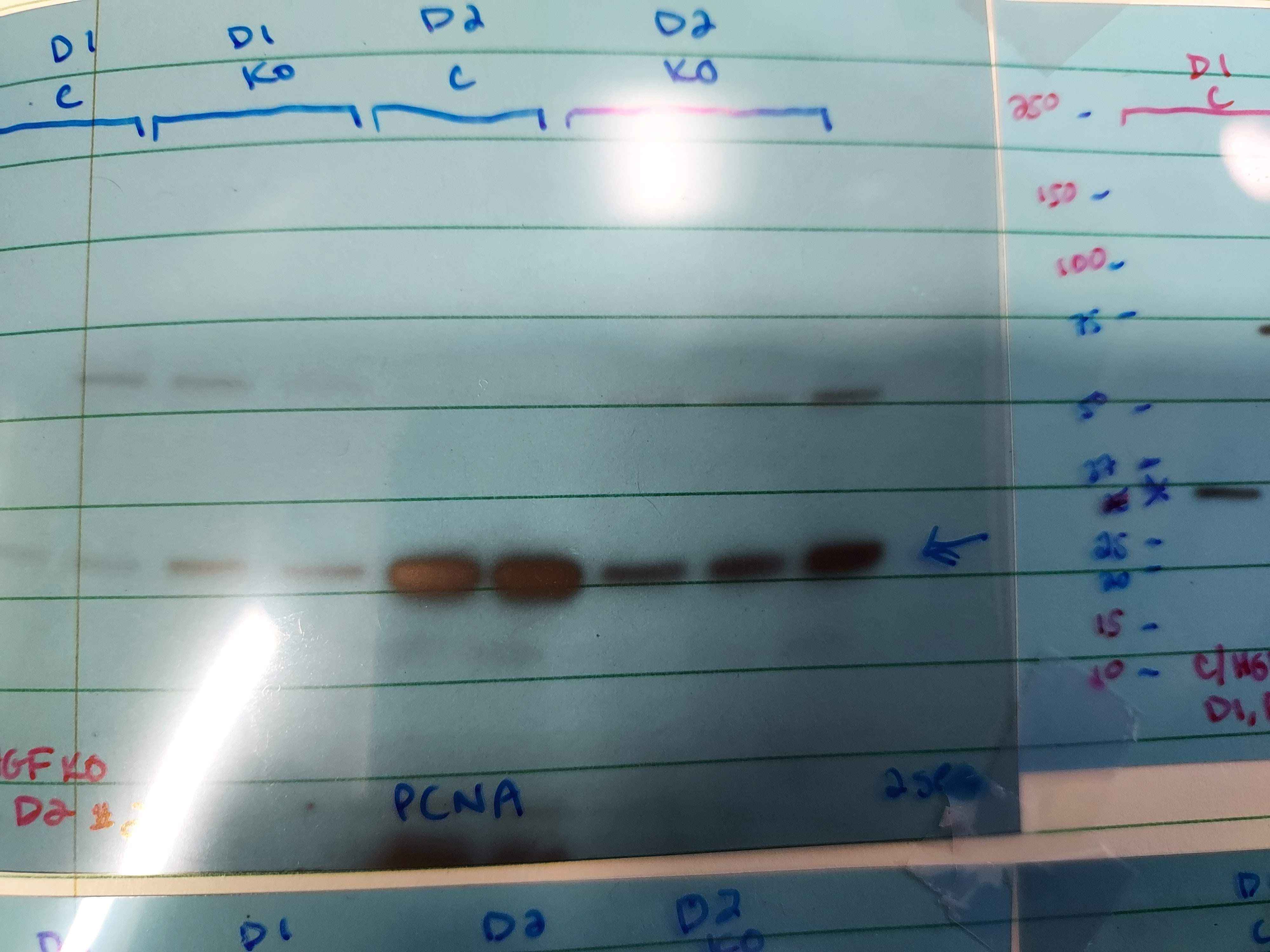

Supplement: S1 File — (ZIP) [file pone.0282358.s001.zip › PLOS ONE images/Fig6E-2.jpg]

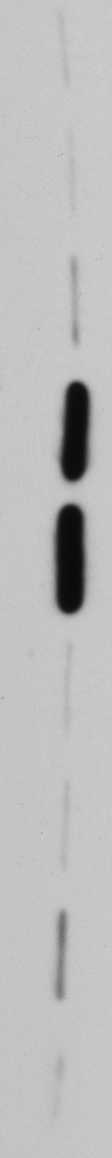

Supplement: S1 File — (ZIP) [file pone.0282358.s001.zip › PLOS ONE images/Fig6E-3.jpg]

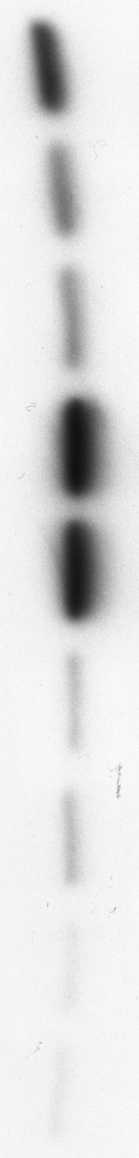

Supplement: S1 File — (ZIP) [file pone.0282358.s001.zip › PLOS ONE images/Fig6E-4.jpg]

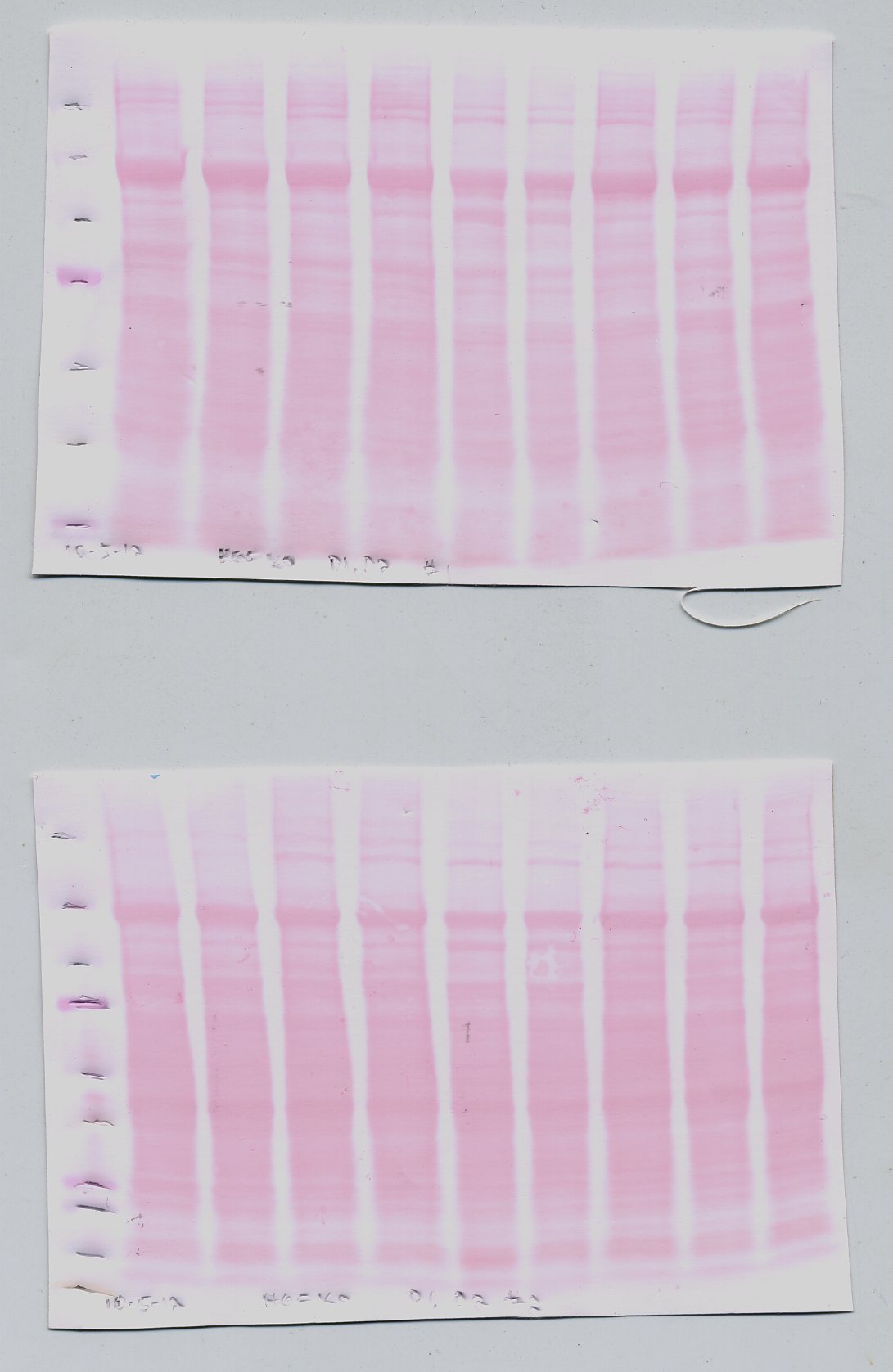

Supplement: S1 File — (ZIP) [file pone.0282358.s001.zip › PLOS ONE images/Fig6Eponceau.jpg]

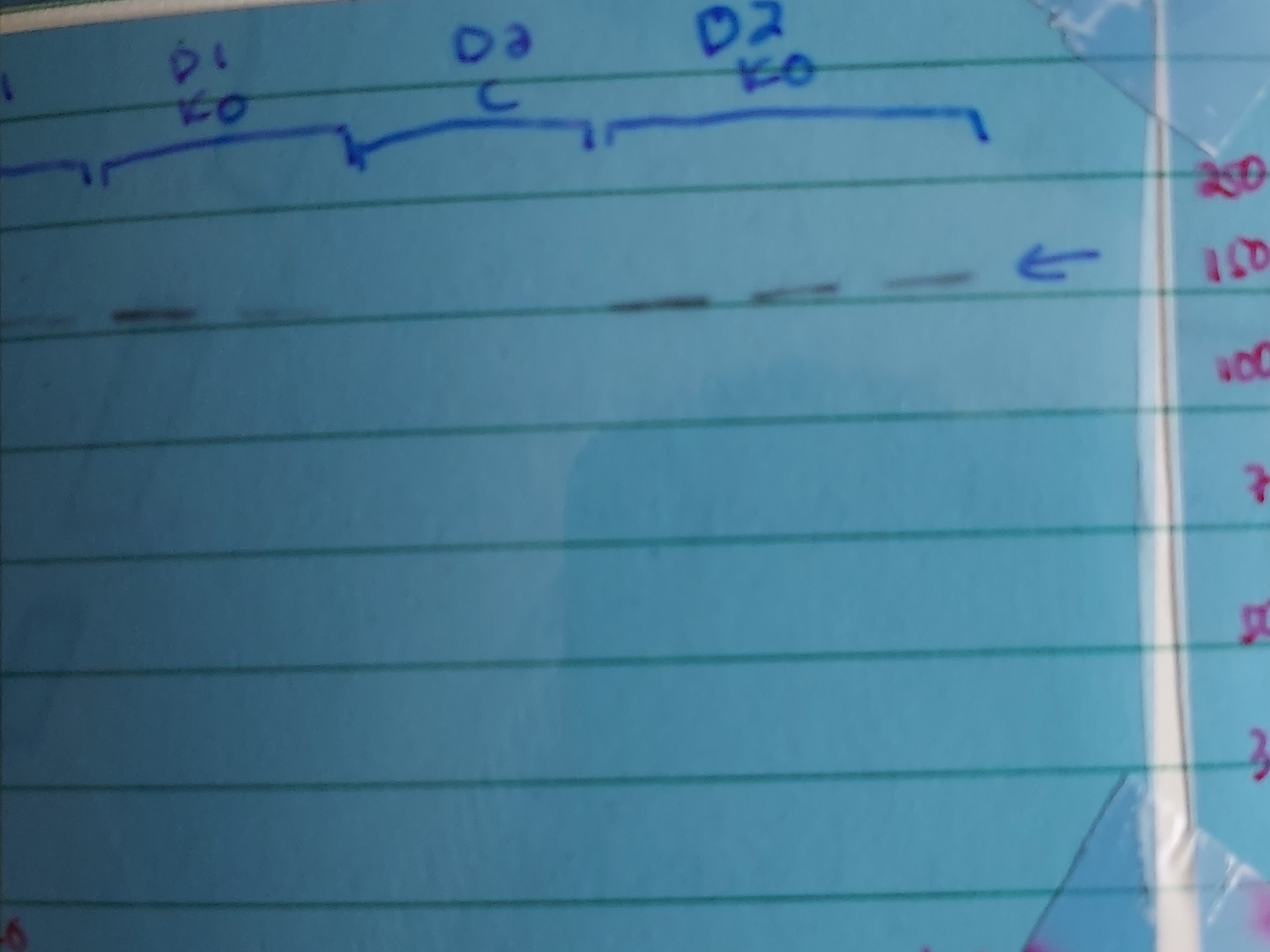

Supplement: S1 File — (ZIP) [file pone.0282358.s001.zip › PLOS ONE images/Fig7A-1.jpg]

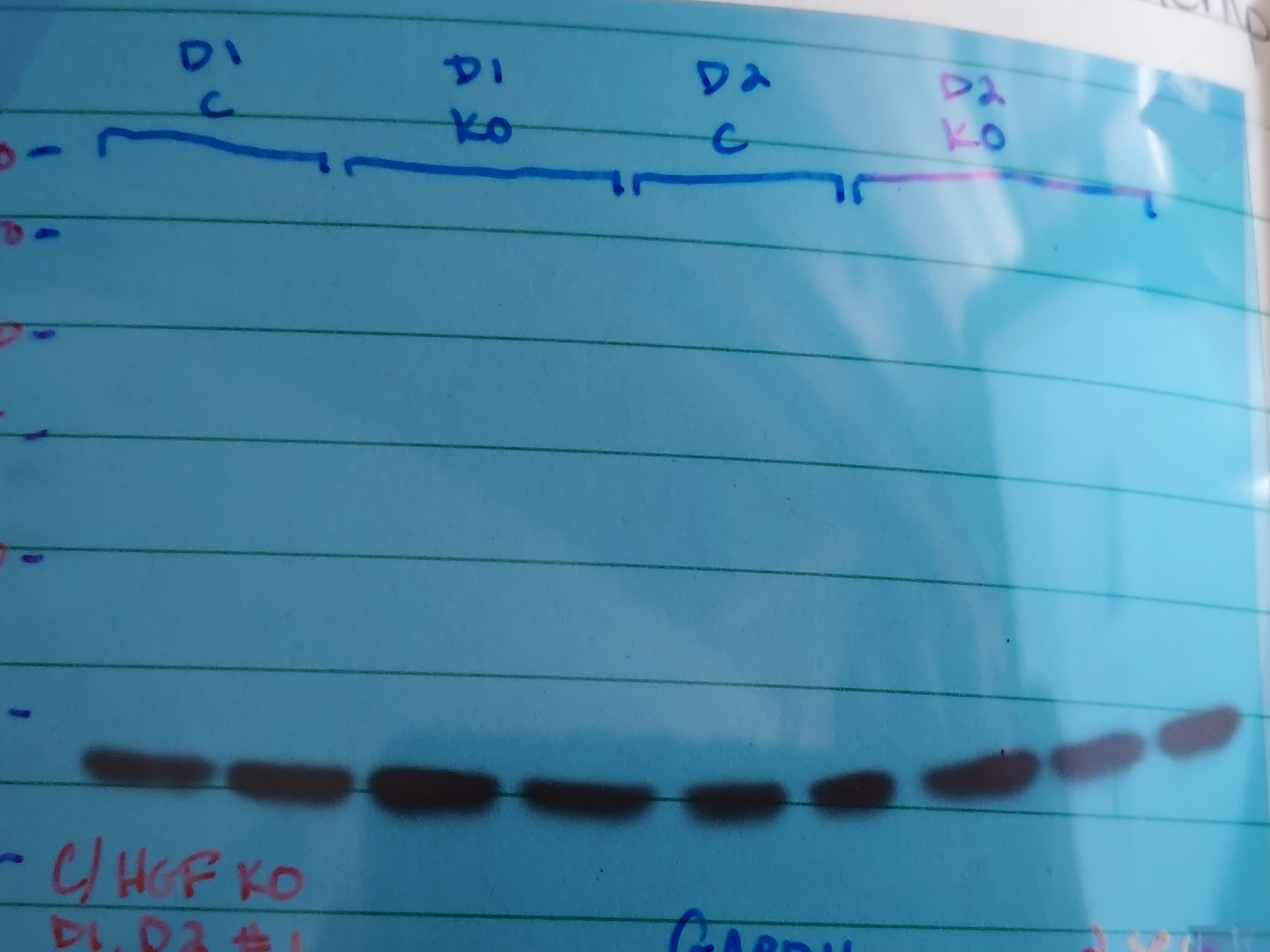

Supplement: S1 File — (ZIP) [file pone.0282358.s001.zip › PLOS ONE images/Fig7A-2.jpg]

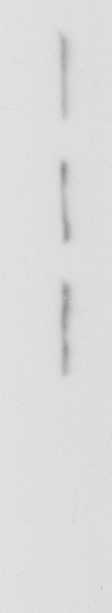

Supplement: S1 File — (ZIP) [file pone.0282358.s001.zip › PLOS ONE images/Fig7A-3.jpg]

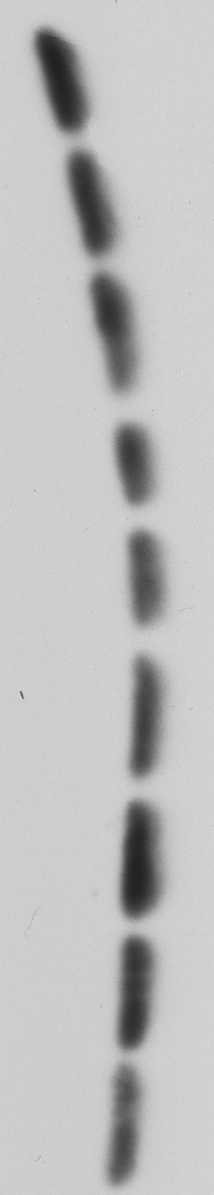

Supplement: S1 File — (ZIP) [file pone.0282358.s001.zip › PLOS ONE images/Fig7A-4.jpg]

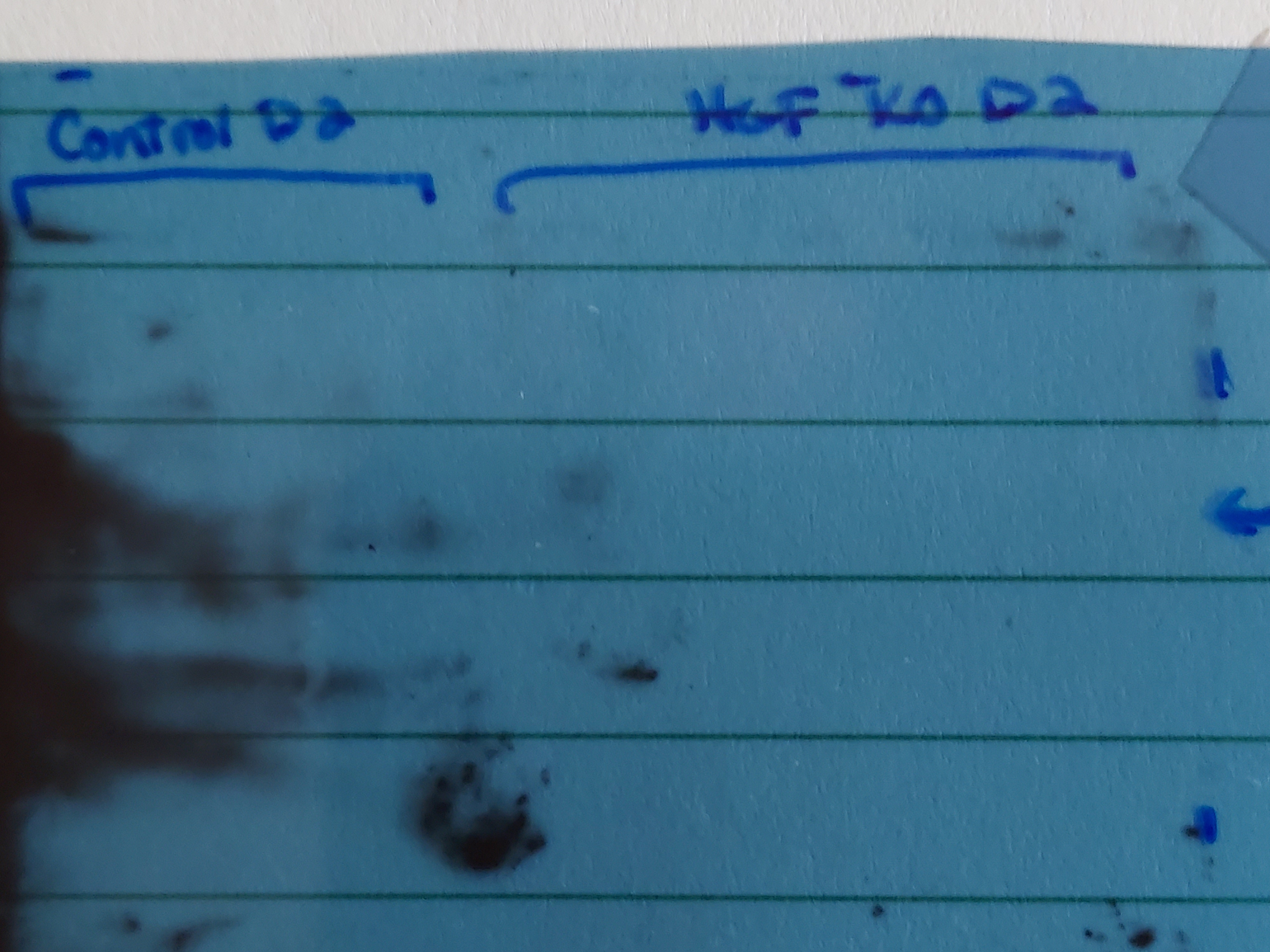

Supplement: S1 File — (ZIP) [file pone.0282358.s001.zip › PLOS ONE images/Fig7B-1.jpg]

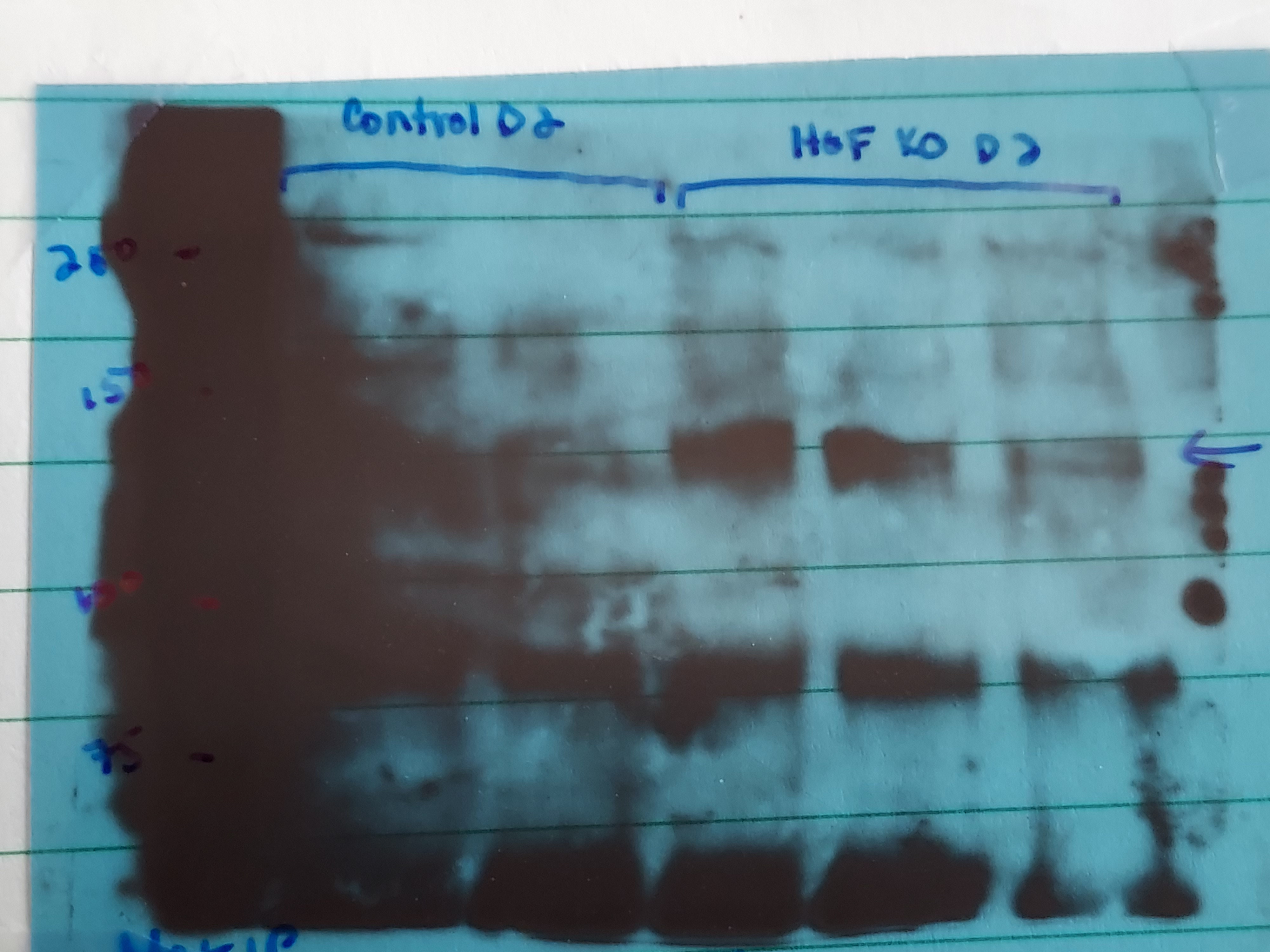

Supplement: S1 File — (ZIP) [file pone.0282358.s001.zip › PLOS ONE images/Fig7B-2.jpg]

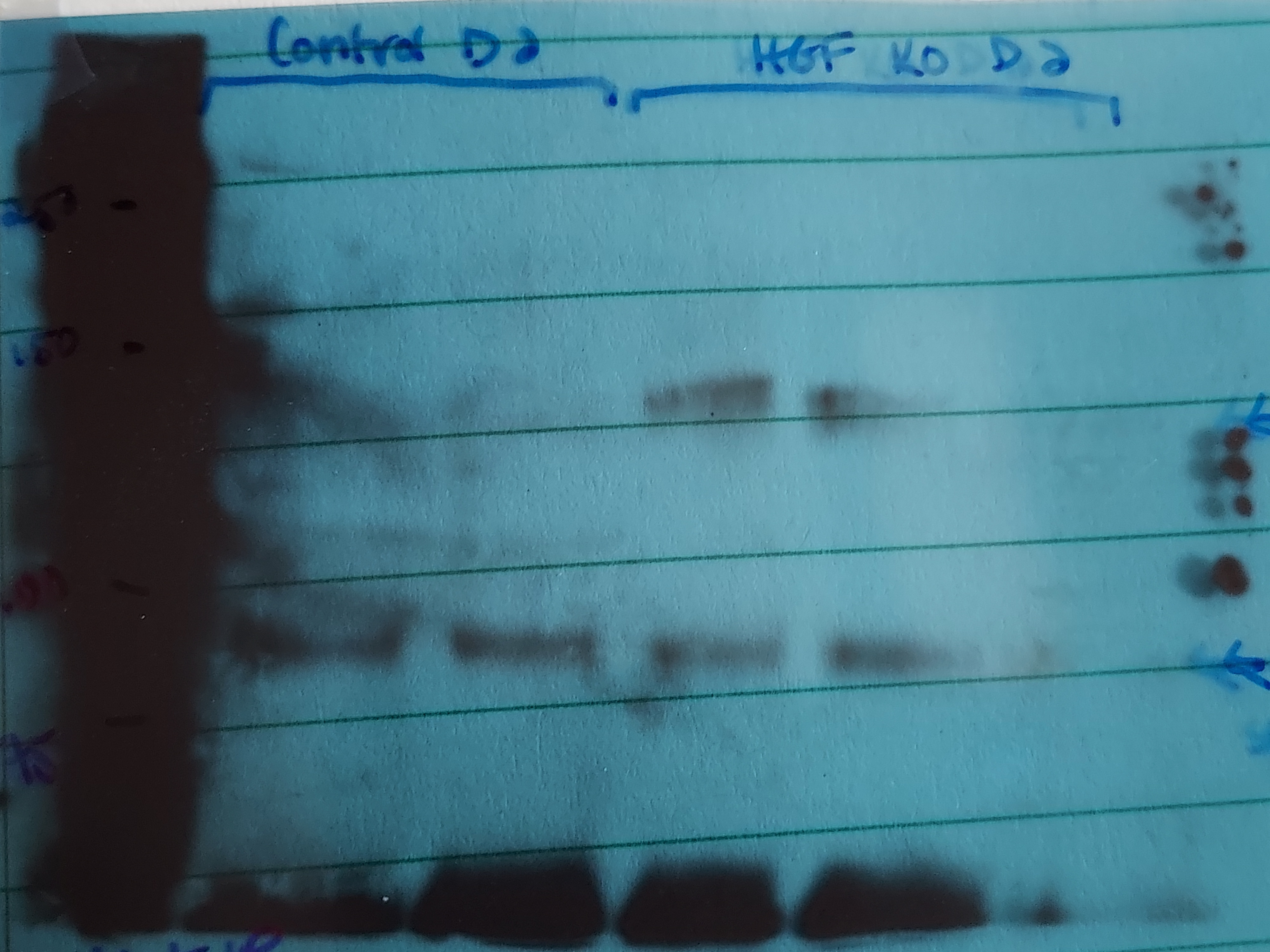

Supplement: S1 File — (ZIP) [file pone.0282358.s001.zip › PLOS ONE images/Fig7B-3.jpg]

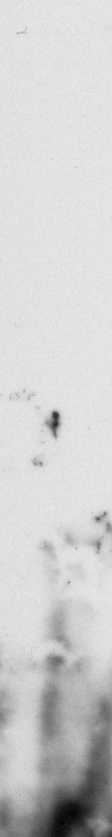

Supplement: S1 File — (ZIP) [file pone.0282358.s001.zip › PLOS ONE images/Fig7B-4.jpg]

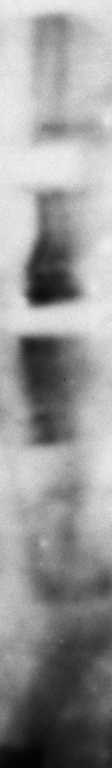

Supplement: S1 File — (ZIP) [file pone.0282358.s001.zip › PLOS ONE images/Fig7B-5.jpg]

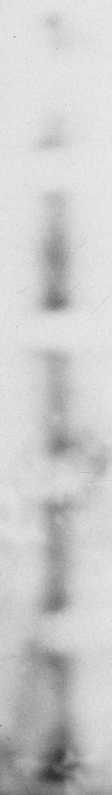

Supplement: S1 File — (ZIP) [file pone.0282358.s001.zip › PLOS ONE images/Fig7B-6.jpg]
